# Evaluating COVID-19 vaccine effectiveness across epidemiologic designs: test-negative case-control versus retrospective cohort analyses in a nationwide study

**DOI:** 10.1093/ije/dyag210

**Published:** 2026-09-22

**Authors:** Layan Sukik, Pradipta Paul, Houssein H Ayoub, Peter Coyle, Patrick Tang, Mohammad R Hasan, Hadi M Yassine, Asmaa A Al Thani, Zaina Al-Kanaani, Einas Al-Kuwari, Andrew Jeremijenko, Anvar Hassan Kaleeckal, Ali Nizar Latif, Riyazuddin Mohammad Shaik, Hanan F Abdul-Rahim, Gheyath K Nasrallah, Mohamed Ghaith Al-Kuwari, Adeel A Butt, Hamad Eid Al-Romaihi, Mohamed H Al-Thani, Abdullatif Al-Khal, Hiam Chemaitelly, Laith J Abu-Raddad

**Affiliations:** Infectious Disease Epidemiology Group, Weill Cornell Medicine-Qatar, Cornell University, Doha, Qatar; Infectious Disease Epidemiology Group, Weill Cornell Medicine-Qatar, Cornell University, Doha, Qatar; Mathematics Program, Department of Mathematics and Statistics, College of Arts and Sciences, Qatar University, Doha, Qatar; Hamad Medical Corporation, Doha, Qatar; Department of Biomedical Science, College of Health Sciences, QU Health, Qatar University, Doha, Qatar; Wellcome-Wolfson Institute for Experimental Medicine, Queens University, Belfast, United Kingdom; Department of Pathology and Laboratory Medicine, University of British Columbia, Vancouver, Canada; Department of Pathology and Molecular Medicine, McMaster University, Hamilton, Canada; Department of Biomedical Science, College of Health Sciences, QU Health, Qatar University, Doha, Qatar; Biomedical Research Center, QU Health, Qatar University, Doha, Qatar; Department of Biomedical Science, College of Health Sciences, QU Health, Qatar University, Doha, Qatar; Biomedical Research Center, QU Health, Qatar University, Doha, Qatar; Hamad Medical Corporation, Doha, Qatar; Hamad Medical Corporation, Doha, Qatar; Hamad Medical Corporation, Doha, Qatar; Hamad Medical Corporation, Doha, Qatar; Hamad Medical Corporation, Doha, Qatar; Hamad Medical Corporation, Doha, Qatar; Department of Public Health, College of Health Sciences, QU Health, Qatar University, Doha, Qatar; Department of Biomedical Science, College of Health Sciences, QU Health, Qatar University, Doha, Qatar; Biomedical Research Center, QU Health, Qatar University, Doha, Qatar; Primary Health Care Corporation, Doha, Qatar; College of Medicine, Qatar University, Doha, Qatar; Hamad Medical Corporation, Doha, Qatar; Department of Medicine, Weill Cornell Medicine, Cornell University, New York, NY, United States; Department of Population Health Sciences, Weill Cornell Medicine, Cornell University, New York, NY, United States; Ministry of Public Health, Doha, Qatar; Ministry of Public Health, Doha, Qatar; Hamad Medical Corporation, Doha, Qatar; Infectious Disease Epidemiology Group, Weill Cornell Medicine-Qatar, Cornell University, Doha, Qatar; Department of Population Health Sciences, Weill Cornell Medicine, Cornell University, New York, NY, United States; Infectious Disease Epidemiology Group, Weill Cornell Medicine-Qatar, Cornell University, Doha, Qatar; Department of Public Health, College of Health Sciences, QU Health, Qatar University, Doha, Qatar; Department of Population Health Sciences, Weill Cornell Medicine, Cornell University, New York, NY, United States; College of Health and Life Sciences, Hamad bin Khalifa University, Doha, Qatar

**Keywords:** SARS-CoV-2, infection, mRNA, efficacy, bias

## Abstract

**Background:**

Observational studies are central to assessing vaccine effectiveness and informing public health policy. The two most common designs are test-negative case-control and cohort studies, yet direct comparisons of their performance using the same population, data sources, and analytic strategies remain rare. This study compares these two designs within a national population using harmonized analytic approaches.

**Methods:**

Nationwide, matched test-negative case-control and retrospective cohort studies were conducted in Qatar between 1 January 2021 and 18 December 2021 to assess the effectiveness of the coronavirus disease (COVID-19) primary series of BNT162b2 and mRNA-1273 against any severe acute respiratory syndrome coronavirus 2 infection, symptomatic infection, and severe COVID-19.

**Results:**

In the test-negative design, BNT162b2 effectiveness was 71.0% [95% confidence interval (CI): 70.1–71.9] against any infection, 75.6% (95% CI: 74.2–77.0) against symptomatic infection, and 96.3% (95% CI: 95.2–97.2) against severe COVID-19. In the cohort design, after adjustment for testing rate, corresponding effectiveness was 78.0% (95% CI: 77.1–78.9), 81.2% (95% CI: 79.8–82.5), and 98.0% (95% CI: 96.6–98.8), respectively. Both designs demonstrated similar, rapid, and largely linear waning of effectiveness in both magnitude and pattern. Test-negative design estimates lay between cohort estimates with and without adjustment for testing rate. Similar results were observed for mRNA-1273.

**Conclusions:**

Both designs yielded consistent effectiveness estimates across vaccines and clinically relevant outcomes, with only modest differences related to handling of health-seeking behavior. These findings demonstrate that, when rigorously implemented using quality data and harmonized analytic strategies, both designs produce comparable effectiveness estimates.

Key Messages

- This study assessed whether test-negative case-control and retrospective cohort designs, when applied to the same population with harmonized data sources and analytic strategies, produce consistent vaccine effectiveness estimates across different clinically relevant outcomes.
- Both designs generated consistent vaccine effectiveness estimates across BNT162b2 and mRNA-1273 and across severe acute respiratory syndrome coronavirus 2 outcomes (any infection, symptomatic infection, and severe coronavirus disease), with similar patterns of rapid waning against infection; modest differences were largely attributable to differential handling of testing behavior.
- The findings provide empirical real-world evidence that, when rigorously implemented with quality data and appropriate control for health-seeking behavior, both designs produce comparable vaccine effectiveness estimates, supporting their use in public health decision-making.

## Introduction

Randomized controlled trials (RCTs) provide high internal validity but may not capture vaccine performance in real-world settings because of selective enrollment, controlled conditions, and limited follow-up [1–3]. Observational studies therefore play a central role in evaluating vaccine effectiveness and informing public health policy, as demonstrated during the coronavirus disease (COVID-19) pandemic [4–6]. Two designs are most commonly used: the test-negative case-control design [6–9] and the retrospective cohort design [6, 10], often implemented within a target trial framework [11, 12]. Each design offers distinct advantages but is subject to specific methodological challenges.

In real-world settings, heterogeneity in health status, healthcare access, and health-seeking behavior can influence both vaccine uptake and detection of infection, thereby affecting vaccine effectiveness estimates [5, 13–15]. Observational estimates may therefore be subject to multiple sources of bias, including confounding, selection bias, and information (misclassification) bias, with their magnitude and direction determined by the study context and design [5, 13, 15, 16]. Despite their widespread use, direct empirical comparisons of the test-negative and retrospective cohort designs within the same population, using harmonized methods, remain limited [16].

In this study, these two approaches were applied to assess the effectiveness of mRNA vaccines against severe acute respiratory syndrome coronavirus 2 (SARS-CoV-2) infection and COVID-19 outcomes within a national population, using aligned analytic strategies to evaluate the consistency of estimates across designs and clinically relevant endpoints. Further background on this study is provided in Supplementary Section 1, in the supplementary material.

## Methods

### Study population and data sources

This study was conducted in the population of Qatar between 1 January 2021, following the launch of the COVID-19 primary series vaccination program [17, 18], and 18 December 2021, the day preceding the onset of the first Omicron wave [19]. The analysis was intentionally restricted to the pre-Omicron period, when estimates were less susceptible to potential confounding or immune imprinting factors arising in later phases of the pandemic [20–24]. Detailed information on the study population and data sources is provided in Supplementary Sections 2–4, in the supplementary material. Infection incidence and circulating variants are presented in Supplementary Fig. S1, in the supplementary material.

### Study design

This article presents two nationwide studies undertaken to enable a rigorous, head-to-head comparison of primary series effectiveness estimates derived from two distinct epidemiologic frameworks: (i) a matched test-negative case-control design and (ii) a matched retrospective cohort design emulating an RCT (target trial design). Effectiveness was evaluated against three endpoints: any SARS-CoV-2 infection, symptomatic infection, and severe COVID-19 (severe [25], critical [25], or fatal [26]).

Vaccine effectiveness was defined as the proportional reduction in susceptibility to infection among vaccinated individuals compared with unvaccinated individuals (reference group) [7, 9, 27]. To ensure internal validity and facilitate direct comparability of estimates, methodological components—including eligibility criteria, exposure classification, outcome definitions, matching variables, follow-up structure, and analytic strategies—were deliberately aligned across both designs.

The exposure, vaccination, was defined as receipt of a two-dose mRNA primary series (BNT162b2 administered 21 days apart and mRNA-1273 administered 28 days apart [28, 29]) with vaccine effects assessed starting 14 days after the second dose to allow for immune development [28, 29]. Outcomes were defined as any SARS-CoV-2 infection, symptomatic infection, and severe, critical, or fatal COVID-19, with standardized definitions for severity [25], criticality [25], and fatality [26] provided in Supplementary Section 5, in the supplementary material. Individuals with documented prior SARS-CoV-2 infection before study entry were excluded in both designs. Calendar time was aligned by implementing both study designs over the same study duration.

Given the large-scale scope and methodological complexity of these investigations, the main text provides a concise overview of the conceptual framework and study design. Detailed methodological documentation is presented in a structured, component-based format in the Supplementary Material. This approach preserves clarity and focus in the article while ensuring comprehensive reporting of all design and analytic elements.

Detailed methods for the test-negative case-control study are provided in Supplementary Section 6, in the supplementary material. Within this framework, effectiveness was estimated by comparing the odds of two-dose primary series vaccination among cases (individuals with SARS-CoV-2-positive tests) and controls (individuals with SARS-CoV-2-negative tests) [7–9, 27]. Detailed methods for the retrospective cohort study are presented in Supplementary Section 7, in the supplementary material. In this design, effectiveness was estimated by comparing the incidence of SARS-CoV-2 infection between individuals who received two vaccine doses (two-dose cohort) and those who remained unvaccinated (unvaccinated cohort) [10, 21, 30].

The retrospective cohort was constructed using the national SARS-CoV-2 testing database rather than a complete population registry. Unvaccinated individuals were identified through linkage of testing and vaccination records, and cohort inclusion required documented testing activity. In contrast, there was no requirement for vaccinated individuals to have a SARS-CoV-2 test during the study. Although this approach differs from classical population register-based cohorts, SARS-CoV-2 testing in Qatar was extensive and largely routine, resulting in coverage of nearly all of the resident population [9, 31]. This is evidenced by the comparable size of the testing database and the national population, with ∼2.7 million individuals tested (Supplementary Figs S2–S5, in the supplementary material) relative to an estimated population size of 2.8 million [32]. This high coverage enabled essentially a population-based study and assessment of SARS-CoV-2 infection outcomes within an actively observed population.

The statistical analysis procedures applied in both studies, including estimation strategies and model specifications, are described in Supplementary Section 8, in the supplementary materials online.

#### Additional analysis

To clarify the theoretical correspondence between estimates derived from the test-negative and cohort designs, an additional analysis applied the test-negative design using the same methodology but restricted to SARS-CoV-2-positive and -negative tests occurring during follow-up within the matched cohorts of the cohort study analyses, thereby nesting the test-negative study within the implemented cohort study. This restriction aligned eligibility criteria and follow-up structure across the two designs, ensuring that both were applied to the same population under observation. To improve statistical precision in this restricted analysis, which was conducted within a smaller matched cohort population, cases were matched to controls at a ratio of up to one-to-five, thereby increasing precision relative to one-to-one matching.

The underlying premise was that restricting the test-negative design to tests occurring within the matched cohort study population would approximate as closely as possible the population, eligibility criteria, and follow-up structure of the cohort design, thereby providing the closest theoretical alignment between the two methodological approaches.

## Results

### Study population

#### Vaccination scale-up

Between 1 January 2021 (study start) and 18 December 2021 (study end), 1 281 243 individuals received at least two doses of BNT162b2 (Supplementary Fig. S6A, in the supplementary material). The median dates of first and second dose administration were 2 May 2021 and 24 May 2021, respectively, with a median inter-dose interval of 21 days [interquartile range (IQR): 21–22 days].

Over the same period, 887 969 individuals received at least two doses of mRNA-1273 (Fig. S6B, in the supplementary material). The median dates of first and second dose administration were 27 May 2021 and 27 June 2021, respectively, with a median inter-dose interval of 28 days (IQR: 28–30 days).

#### Test-negative case-control study

The population selection process for the BNT162b2 analysis is shown in Fig. S2, in the supplementary material. Baseline characteristics of the eligible unmatched groups are summarized in Supplementary Table S1, in the supplementary material, and characteristics of the matched groups are presented in Table 1. Corresponding information for the mRNA-1273 analysis is provided in Supplementary Fig. S3 and Supplementary Table S1, in the supplementary material, and Table 1.

**Table 1:** Characteristics of the matched cases and controls in the BNT162b2 and mRNA-1273 analyses of the test-negative case-control study.

Characteristics	BNT162b2 analysis			mRNA-1273 analysis		
	Cases^a^	Controls^a^	SMD^b^	Cases^a^	Controls^a^	SMD^b^
	*N* = 200 541	*N* = 200 541		*N* = 186 968	*N* = 186 968	
Median age (IQR), years	32 (24–39)	32 (24–39)	0.00^c^	31 (24–38)	31 (24–38)	0.00^c^
Age group, *n* (%)						
<10 years	19 473 (9.7)	19 473 (9.7)	0.00	19 479 (10.4)	19 479 (10.4)	0.00
10–19 years	17 757 (8.9)	17 757 (8.9)		16 683 (8.9)	16 683 (8.9)	
20–29 years	45 975 (22.9)	45 975 (22.9)		44 195 (23.6)	44 195 (23.6)	
30–39 years	70 444 (35.1)	70 444 (35.1)		66 200 (35.4)	66 200 (35.4)	
40–49 years	33 962 (16.9)	33 962 (16.9)		30 585 (16.4)	30 585 (16.4)	
50–59 years	10 158 (5.1)	10 158 (5.1)		8284 (4.4)	8284 (4.4)	
60–69 years	2221 (1.1)	2221 (1.1)		1281 (0.7)	1281 (0.7)	
70+ years	551 (0.3)	551 (0.3)		261 (0.1)	261 (0.1)	
Sex						
Male	136 979 (68.3)	136 979 (68.3)	0.00	129 050 (69.0)	129 050 (69.0)	0.00
Female	63 562 (31.7)	63 562 (31.7)		57 918 (31.0)	57 918 (31.0)	
Nationality^d^						
Bangladeshi	14 069 (7.0)	14 069 (7.0)	0.00	13 789 (7.4)	13 789 (7.4)	0.00
Egyptian	11 522 (5.7)	11 522 (5.7)		10 240 (5.5)	10 240 (5.5)	
Filipino	22 432 (11.2)	22 432 (11.2)		21 443 (11.5)	21 443 (11.5)	
Indian	57 126 (28.5)	57 126 (28.5)		54 948 (29.4)	54 948 (29.4)	
Nepalese	16 695 (8.3)	16 695 (8.3)		16 555 (8.9)	16 555 (8.9)	
Pakistani	9746 (4.9)	9746 (4.9)		9321 (5.0)	9321 (5.0)	
Qatari	25 851 (12.9)	25 851 (12.9)		21 032 (11.2)	21 032 (11.2)	
Sri Lankan	6103 (3.0)	6103 (3.0)		5985 (3.2)	5985 (3.2)	
Sudanese	4963 (2.5)	4963 (2.5)		4593 (2.5)	4593 (2.5)	
Other nationalities^e^	32 034 (16.0)	32 034 (16.0)		29 062 (15.5)	29 062 (15.5)	
Coexisting conditions						
0	167 889 (83.7)	167 889 (83.7)	0.00	159 827 (85.5)	159 827 (85.5)	0.00
1	20 738 (10.3)	20 738 (10.3)		18 339 (9.8)	18 339 (9.8)	
2	7256 (3.6)	7256 (3.6)		5911 (3.2)	5911 (3.2)	
3	2347 (1.2)	2347 (1.2)		1598 (0.9)	1598 (0.9)	
4	1075 (0.5)	1075 (0.5)		648 (0.3)	648 (0.3)	
5	581 (0.3)	581 (0.3)		309 (0.2)	309 (0.2)	
6+	655 (0.3)	655 (0.3)		336 (0.2)	336 (0.2)	
Reason for testing						
Clinical suspicion	55 327 (27.6)	55 327 (27.6)	0.00	50 320 (26.9)	50 320 (26.9)	0.00
Contact tracing	23 289 (11.6)	23 289 (11.6)		22 267 (11.9)	22 267 (11.9)	
Port of entry	45 890 (22.9)	45 890 (22.9)		43 848 (23.5)	43 848 (23.5)	
Individual request	14 910 (7.4)	14 910 (7.4)		13 994 (7.5)	13 994 (7.5)	
Survey	34 618 (17.3)	34 618 (17.3)		32 306 (17.3)	32 306 (17.3)	
Healthcare routine testing	17 795 (8.9)	17 795 (8.9)		17 319 (9.3)	17 319 (9.3)	
Pre-travel	8430 (4.2)	8430 (4.2)		6677 (3.6)	6677 (3.6)	
Other	282 (0.1)	282 (0.1)		237 (0.1)	237 (0.1)	

Abbreviations: IQR, interquartile range; SARS-CoV-2, severe acute respiratory syndrome coronavirus 2; SMD, standardized mean difference.

a Cases represent SARS-CoV-2-positive tests and controls represent SARS-CoV-2-negative tests. Cases and controls were matched exactly one-to-one by sex, 10-year age group, nationality, number of coexisting conditions, calendar week of testing, and reason for testing.

b SMD is the difference in the mean of a covariate between groups divided by the pooled standard deviation. An SMD of ≤0.1 indicates adequate matching.

c SMD is for the mean difference between groups divided by the pooled standard deviation.

d Nationalities were chosen to represent the most populous groups in Qatar.

e These comprise up to 125 and 121 other nationalities in Qatar in the BNT162b2 and mRNA-1273 analyses, respectively.

This national-level study provides findings that are broadly representative of the country’s internationally diverse population, characterized by a predominantly young age distribution and a male majority.

#### Retrospective cohort study

The cohort selection process for the BNT162b2 analysis is presented in Fig. S4, in the supplementary material. Characteristics of the eligible unmatched cohorts are summarized in Supplementary Table S2, in the supplementary material, while characteristics of the matched cohorts are shown in Table 2. Corresponding information for the mRNA-1273 analysis is provided in Supplementary Fig. S5 and Supplementary Table S2, in the supplementary material, and Table 2.

**Table 2:** Baseline characteristics of the matched vaccinated and unvaccinated cohorts in the BNT162b2 and mRNA-1273 analyses of the retrospective cohort study.

Characteristics	BNT162b2 analysis			mRNA-1273 analysis		
	Two-dose cohort^a^	Unvaccinated cohort^a^	SMD^b^	Two-dose cohort^a^	Unvaccinated cohort^a^	SMD^b^
	*N* = 536 788	*N* = 536 788		*N* = 344 797	*N* = 344 797	
**Median age (IQR), years**	36 (29–42)	34 (28–42)	0.08^c^	36 (30–41)	35 (30–41)	0.08^c^
**Age group, *n* (%)**						
<10 years	5 (0.0)	5 (0.0)	0.00	0 (0.0)	0 (0.0)	0.00
10–19 years	51 047 (9.5)	51 047 (9.5)		12 (0.0)	12 (0.0)	
20–29 years	99 666 (18.6)	99 666 (18.6)		75 584 (21.9)	75 584 (21.9)	
30–39 years	211 927 (39.5)	211 927 (39.5)		163 963 (47.6)	163 963 (47.6)	
40–49 years	118 337 (22.0)	118 337 (22.0)		75 650 (21.9)	75 650 (21.9)	
50–59 years	41 887 (7.8)	41 887 (7.8)		23 759 (6.9)	23 759 (6.9)	
60–69 years	10 889 (2.0)	10 889 (2.0)		4943 (1.4)	4943 (1.4)	
70+ years	3030 (0.6)	3030 (0.6)		886 (0.3)	886 (0.3)	
**Sex**						
Male	381 464 (71.1)	381 464 (71.1)	0.00	275 940 (80.0)	275 940 (80.0)	0.00
Female	155 324 (28.9)	155 324 (28.9)		68 857 (20.0)	68 857 (20.0)	
**Nationality** ^d^						
Bangladeshi	43 630 (8.1)	43 630 (8.1)	0.00	35 418 (10.3)	35 418 (10.3)	0.00
Egyptian	29 822 (5.6)	29 822 (5.6)		12 945 (3.8)	12 945 (3.8)	
Filipino	49 276 (9.2)	49 276 (9.2)		28 637 (8.3)	28 637 (8.3)	
Indian	161 841 (30.1)	161 841 (30.1)		132 843 (38.5)	132 843 (38.5)	
Nepalese	42 300 (7.9)	42 300 (7.9)		38 191 (11.1)	38 191 (11.1)	
Pakistani	30 280 (5.6)	30 280 (5.6)		22 650 (6.6)	22 650 (6.6)	
Qatari	52 673 (9.8)	52 673 (9.8)		10 307 (3.0)	10 307 (3.0)	
Sri Lankan	14 492 (2.7)	14 492 (2.7)		10 543 (3.1)	10 543 (3.1)	
Sudanese	13 270 (2.5)	13 270 (2.5)		6624 (1.9)	6624 (1.9)	
Other nationalities^e^	99 204 (18.5)	99 204 (18.5)		46 639 (13.5)	46 639 (13.5)	
**Coexisting conditions**						
0	466 772 (87.0)	466 772 (87.0)	0.00	321 541 (93.3)	321 541 (93.3)	0.00
1	40 355 (7.5)	40 355 (7.5)		13 994 (4.1)	13 994 (4.1)	
2	15 948 (3.0)	15 948 (3.0)		5481 (1.6)	5481 (1.6)	
3	6474 (1.2)	6474 (1.2)		1964 (0.6)	1964 (0.6)	
4	3356 (0.6)	3356 (0.6)		906 (0.3)	906 (0.3)	
5	1824 (0.3)	1824 (0.3)		433 (0.1)	433 (0.1)	
6+	2059 (0.4)	2059 (0.4)		478 (0.1)	478 (0.1)	

Abbreviations: IQR, interquartile range; SARS-CoV-2, severe acute respiratory syndrome coronavirus 2; SMD, standardized mean difference.

a Cohorts were matched exactly one-to-one by sex, 10-year age group, nationality, and number of coexisting conditions. Individuals who received their second dose in a specific calendar week in the two-dose cohort were additionally matched to individuals who had a record for a SARS-CoV-2-negative test in that same calendar week in the unvaccinated cohort, to ensure that matched pairs had presence in Qatar over the same time period.

b SMD is the difference in the mean of a covariate between groups divided by the pooled standard deviation. An SMD of ≤0.1 indicates adequate matching.

c SMD is for the mean difference between groups divided by the pooled standard deviation.

d Nationalities were chosen to represent the most populous groups in Qatar.

e These comprise up to 141 and 133 other nationalities in Qatar in the BNT162b2 and mRNA-1273 analyses, respectively.

This national study yields findings broadly representative of Qatar’s diverse population, characterized by a predominantly young, male demographic.

### Vaccine effectiveness

#### Test-negative case-control study

**Figure 1:**
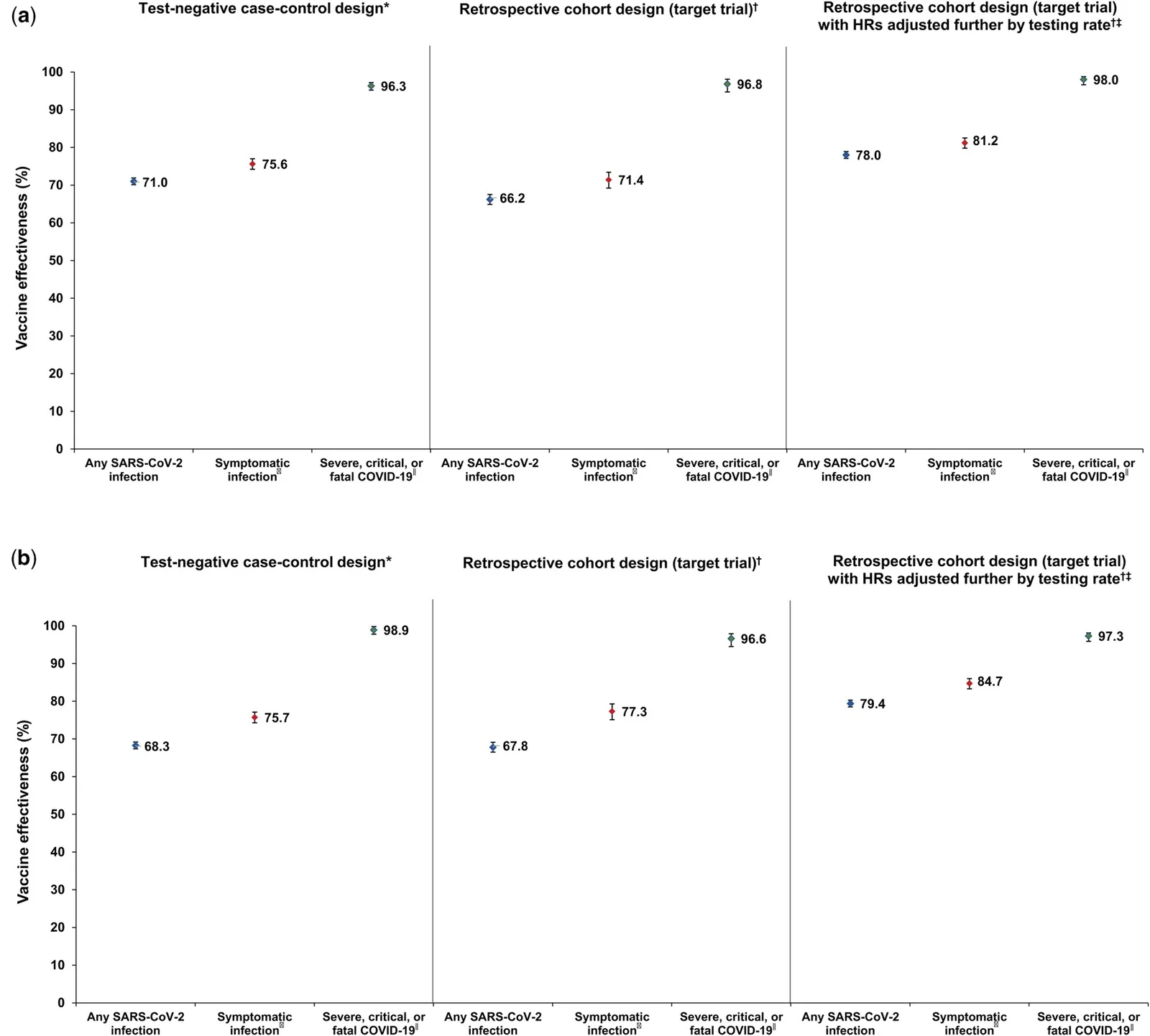
Comparison of vaccine effectiveness estimates for the primary series of (a) BNT162b2 and (b) mRNA-1273 across the test-negative case-control design, retrospective cohort design without adjustment for testing rate, and retrospective cohort design with adjustment for testing rate. Effectiveness is shown against any SARS-CoV-2 infection, symptomatic infection, and severe, critical, or fatal COVID-19. COVID-19, coronavirus disease; SARS-CoV-2, severe acute respiratory syndrome coronavirus 2. *Cases and controls were matched exactly one-to-one for any SARS-CoV-2 infection and symptomatic infection, and one-to-five for severe, critical, or fatal COVID-19. Not all cases in the severity outcome could be matched to exactly five eligible controls; all matched sets with at least one eligible control were retained in the analysis. Matching was done by sex, 10-year age group, nationality, number of coexisting conditions, calendar week of testing, and reason for testing. Vaccine effectiveness estimates were based on odds ratios obtained from conditional logistic regressions. ^†^Cohorts were matched exactly one-to-one by sex, 10-year age group, nationality, and number of coexisting conditions. Individuals who received their second dose in a specific calendar week in the two-dose cohort were additionally matched to individuals who had a record for a SARS-CoV-2-negative test in that same calendar week in the unvaccinated cohort, to ensure that matched pairs had presence in Qatar over the same time period. Vaccine effectiveness estimates were derived from hazard ratios adjusted for sex, 10-year age group, nationality groups, number of coexisting conditions, and calendar week of the second dose in the two-dose cohort or of the SARS-CoV-2-negative test in the unvaccinated cohort. ^‡^Vaccine effectiveness estimates were derived from hazard ratios that were additionally adjusted for testing rate. ^§^A symptomatic infection was defined as a SARS-CoV-2-positive test conducted because of the presence of symptoms consistent with a respiratory tract infection. ^**‖**^Severity, criticality, and fatality were defined according to the World Health Organization guidelines.

Vaccine effectiveness of the two-dose primary BNT162b2 series against any SARS-CoV-2 infection was 71.0% [95% confidence interval (CI): 70.1–71.9], with a median interval of 83 days (IQR: 36–163) between receipt of the second dose and the testing date (Table 3 and Fig. 1).

**Figure 2:**
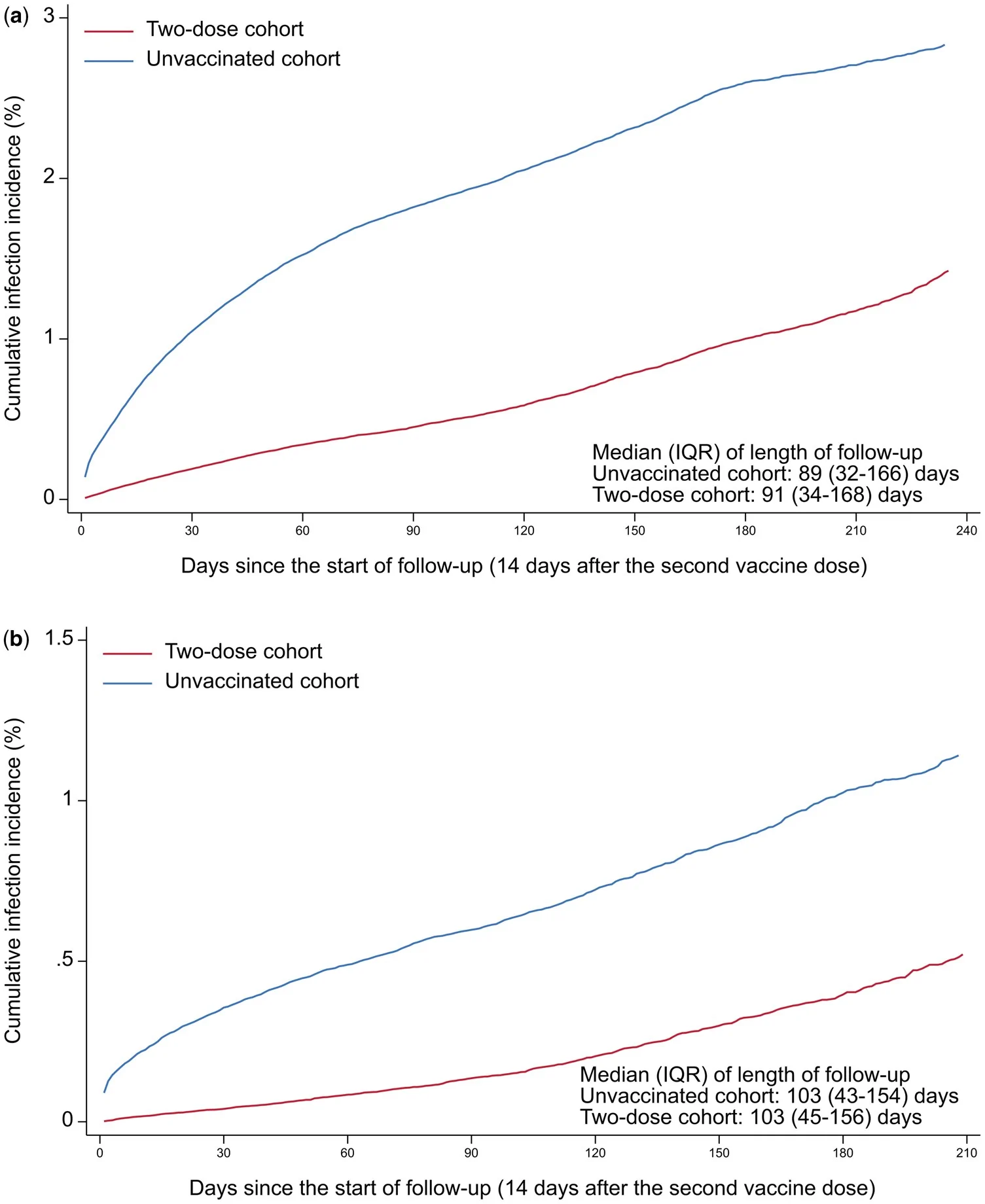
Cumulative incidence of SARS-CoV-2 infection in the matched vaccinated and unvaccinated cohorts in the (a) BNT162b2 and (b) mRNA-1273 analyses of the retrospective cohort study. IQR, interquartile range; SARS-CoV-2, severe acute respiratory syndrome coronavirus 2. The *x*-axis scale reflects the available follow-up time, which was longer for BNT162b2 than for mRNA-1273 because the roll-out of BNT162b2 began ∼3 months earlier.

**Figure 3:**
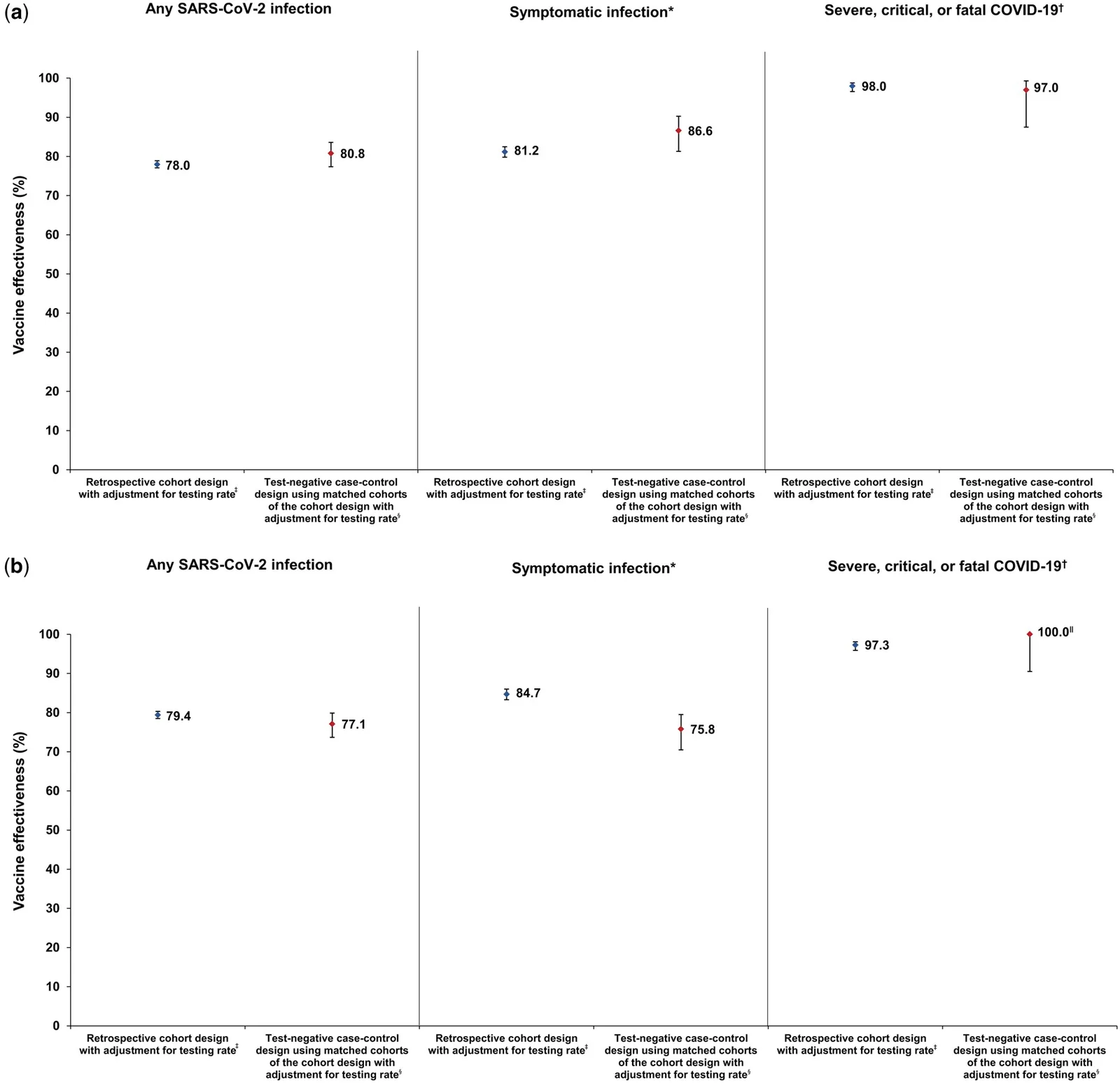
Additional analysis restricting the test-negative design to SARS-CoV-2-positive and -negative tests occurring during cohort study follow-up for (a) BNT162b2 and (b) mRNA-1273. Effectiveness is shown against any SARS-CoV-2 infection, symptomatic infection, and severe, critical, or fatal COVID-19. COVID-19, coronavirus disease; SARS-CoV-2, severe acute respiratory syndrome coronavirus 2. *A symptomatic infection was defined as a SARS-CoV-2-positive test conducted because of the presence of symptoms consistent with a respiratory tract infection. †Severity, criticality, and fatality were defined according to the World Health Organization guidelines. ‡Cohorts were matched exactly one-to-one by sex, 10-year age group, nationality, and number of coexisting conditions. Individuals who received their second dose in a specific calendar week in the two-dose cohort were additionally matched to individuals who had a record for a SARS-CoV-2-negative test in that same calendar week in the unvaccinated cohort, to ensure that matched pairs had presence in Qatar over the same time period. Vaccine effectiveness estimates were derived from hazard ratios adjusted for sex, 10-year age group, nationality groups, number of coexisting conditions, calendar week of the second dose in the two-dose cohort or of the SARS-CoV-2-negative test in the unvaccinated cohort, and testing rate. ^§^Cases and controls were selected from matched samples of the retrospective cohort design and classified as cases or controls according to positive or negative test results during follow-up. Cases and controls were matched exactly one-to-five to increase statistical power, given the low number of events. Not all cases could be matched to exactly five eligible controls; all matched sets with at least one eligible control were retained in the analysis. Matching was done by sex, 10-year age group, nationality, number of coexisting conditions, calendar week of testing, and reason for testing. Vaccine effectiveness estimates were based on odds ratios obtained from conditional logistic regressions. ^‖^The 95% CI was estimated with the use of McNemar’s test because of zero events among exposed cases.

**Table 3:** Effectiveness of primary-series BNT162b2 and mRNA-1273 vaccination against any SARS-CoV-2 infection, symptomatic infection, and severe, critical, or fatal COVID-19 in the test-negative case-control study.

Effectiveness against	Cases (SARS-CoV-2-positive tests)		Controls (SARS-CoV-2-negative tests)		Effectiveness % (95% CI)
	Vaccinated	Unvaccinated	Vaccinated	Unvaccinated	
**BNT162b2 analysis**					
Any SARS-CoV-2 infection^a^	13 557	186 984	26 026	174 515	71.0 (70.1–71.9)
Symptomatic infection^a^^,^^b^	3942	51 385	8246	47 081	75.6 (74.2–77.0)
Severe, critical, or fatal COVID-19^c^	115	4728	3535	16 735	96.3 (95.2–97.2)
**mRNA-1273 analysis**					
Any SARS-CoV-2 infection^a^	2550	184 418	5551	181 417	68.3 (66.3–70.2)
Symptomatic infection^a^^,^^b^	465	49 855	1163	49 157	75.7 (71.9–79.0)
Severe, critical, or fatal COVID-19^c^	3	4453	419	18 052	98.9 (95.5–99.7)

Abbreviations: CI, confidence interval; COVID-19, coronavirus disease; SARS-CoV-2, severe acute respiratory syndrome coronavirus 2.

a Cases and controls were matched exactly one-to-one by sex, 10-year age group, nationality, number of coexisting conditions, calendar week of testing, and reason for testing.

b A symptomatic infection was defined as a SARS-CoV-2-positive test conducted because of the presence of symptoms consistent with a respiratory tract infection.

c Cases and controls were matched exactly one-to-five by sex, 10-year age group, nationality, number of coexisting conditions, calendar week of testing, and reason for testing. Not all cases could be matched to exactly five eligible controls; all matched sets with at least one eligible control were retained in the analysis. Severity, criticality, and fatality were defined according to the World Health Organization guidelines.

BNT162b2 effectiveness against symptomatic infection was 75.6% (95% CI: 74.2–77.0), with a median interval of 71 days (IQR: 34–158) between receipt of the second dose and the testing date (Table 3 and Fig. 1). BNT162b2 effectiveness against severe, critical, or fatal COVID-19 was 96.3% (95% CI: 95.2–97.2), with a median interval of 46 days (IQR: 27–84) between receipt of the second dose and the testing date.

Vaccine effectiveness of the two-dose primary mRNA-1273 series against any SARS-CoV-2 infection was 68.3% (95% CI: 66.3–70.2), with a median interval of 105 days (IQR: 51–155) between receipt of the second dose and the testing date (Table 3 and Fig. 1).mRNA-1273 effectiveness against symptomatic infection was 75.7% (95% CI: 71.9–79.0), with a median interval of 98.5 days (IQR: 46–158.5) between receipt of the second dose and the testing date (Table 3 and Fig. 1). mRNA-1273 effectiveness against severe, critical, or fatal COVID-19 was 98.9% (95% CI: 95.5–99.7), with a median interval of 67 days (IQR: 31–132) between receipt of the second dose and the testing date.

Effectiveness assessed at 3-month intervals following vaccination showed rapid waning of protection against any and symptomatic infection for both vaccines, whereas protection against severe, critical, or fatal COVID-19 declined only very modestly over time (Fig. S7, in the supplementary material).

#### Retrospective cohort study

The cumulative incidence of SARS-CoV-2 infection for the matched two-dose and unvaccinated cohorts is shown in Fig. 2a for the BNT162b2 analysis and Fig. 2b for the mRNA-1273 analysis. Median follow-up was 89 days (IQR: 32–166) for the unvaccinated cohort and 91 days (IQR: 34–168) for the two-dose cohort in the BNT162b2 analysis, and 103 days (IQR: 43–154) for the unvaccinated cohort and 103 days (IQR: 45–156) for the two-dose cohort in the mRNA-1273 analysis.

Testing rates differed by vaccination status. In the BNT162b2 analysis, mean testing rates were 1.8 tests per person-year for the unvaccinated cohort and 3.1 tests per person-year for the two-dose cohort. Corresponding rates in the mRNA-1273 analysis were 1.2 and 1.8 tests per person-year, respectively.

Vaccine effectiveness of the two-dose primary BNT162b2 series against any SARS-CoV-2 infection was 66.2% (95% CI: 64.9–67.5) before adjustment for testing rate and increased to 78.0% (95% CI: 77.1–78.9) after adjustment (Table 4 and Fig. 1).

**Table 4:** Effectiveness of primary-series BNT162b2 and mRNA-1273 vaccination against any SARS-CoV-2 infection, symptomatic infection, and severe, critical, or fatal COVID-19 in the retrospective cohort design, with and without adjustment for testing rate.

A) BNT162b2 analysis	**Two-dose cohort** ^a^	**Unvaccinated cohort** ^a^
Sample size	536 788	536 788
Number of any incident infections	3256	9432
Number of incident symptomatic infections	939	3199
Number of severe, critical, or fatal COVID-19^b^ infections	15	465
Incidence rate of SARS-CoV-2 infection (per 10 000 person- weeks; 95% CI)	4.0 (3.9–4.2)	11.9 (11.6–12.1)
Total follow-up time (person-weeks)	8 048 507	7 943 588
**Any SARS-CoV-2 infection**		
Unadjusted hazard ratio (95% CI)	0.34 (0.33–0.36)	
Adjusted hazard ratio (95% CI)^c^	0.34 (0.32–0.35)	
Vaccine effectiveness (95% CI)^d^	66.2 (64.9–67.5)	
Adjusted hazard ratio including further adjustment by testing rate (95% CI)^e^	0.22 (0.21–0.23)	
Vaccine effectiveness (95% CI)^f^	78.0 (77.1–78.9)	
**Symptomatic infection**		
Unadjusted hazard ratio (95% CI)	0.29 (0.27–0.31)	
Adjusted hazard ratio (95% CI)^c^	0.29 (0.27–0.31)	
Vaccine effectiveness (95% CI)^d^	71.4 (69.2–73.4)	
Adjusted hazard ratio including further adjustment by testing rate (95% CI)^e^	0.19 (0.17–0.20)	
Vaccine effectiveness (95% CI)^f^	81.2 (79.8–82.5)	
**Severe, critical, or fatal COVID-19**^b^		
Unadjusted hazard ratio (95% CI)	0.03 (0.02–0.05)	
Adjusted hazard ratio (95% CI)^c^	0.03 (0.02–0.05)	
Vaccine effectiveness (95% CI)^d^	96.8 (94.7–98.1)	
Adjusted hazard ratio including further adjustment by testing rate (95% CI)^e^	0.02 (0.01–0.03)	
Vaccine effectiveness (95% CI)^f^	98.0 (96.6–98.8)	
**B) mRNA-1273 analysis**	**Two-dose cohort** ^a^	**Unvaccinated cohort** ^a^
Sample size	344 797	344 797
Number of any incident infections	741	2285
Number of incident symptomatic infections	141	615
Number of severe, critical, or fatal COVID-19^b^ infections	3	86
Incidence rate of SARS-CoV-2 infection (per 10 000 person- weeks; 95% CI)	1.4 (1.3–1.5)	4.5 (4.3–4.6)
Total follow-up time (person-weeks)	5 158 128	5 128 914
**Any SARS-CoV-2 infection**		
Unadjusted hazard ratio (95% CI)	0.32 (0.30–0.35)	
Adjusted hazard ratio (95% CI)^c^	0.32 (0.30–0.35)	
Vaccine effectiveness (95% CI)^d^	67.8 (65.1–70.4)	
Adjusted hazard ratio including further adjustment by testing rate (95% CI)^e^	0.21 (0.19–0.22)	
Vaccine effectiveness (95% CI)^f^	79.4 (77.6–81.0)	
**Symptomatic infection**		
Unadjusted hazard ratio (95% CI)	0.23 (0.19–0.27)	
Adjusted hazard ratio (95% CI)^c^	0.23 (0.19–0.27)	
Vaccine effectiveness (95% CI)^d^	77.3 (72.8–81.1)	
Adjusted hazard ratio including further adjustment by testing rate (95% CI)^e^	0.15 (0.13–0.18)	
Vaccine effectiveness (95% CI)^f^	84.7 (81.6–87.3)	
**Severe, critical, or fatal COVID-19**^b^		
Unadjusted hazard ratio (95% CI)	0.03 (0.01–0.11)	
Adjusted hazard ratio (95% CI)^c^	0.03 (0.01–0.11)	
Vaccine effectiveness (95% CI)^d^	96.6 (89.2–98.9)	
Adjusted hazard ratio including further adjustment by testing rate (95% CI)^e^	0.03 (0.01–0.09)	
Vaccine effectiveness (95% CI)^f^	97.3 (91.4–99.1)	

Abbreviations: CI, confidence interval; COVID-19, coronavirus disease; SARS-CoV-2, severe acute respiratory syndrome coronavirus 2.

a Cohorts were matched exactly one-to-one by sex, 10-year age group, nationality, and number of coexisting conditions. Individuals who received their second dose in a specific calendar week in the two-dose cohort were additionally matched to individuals who had a record for a SARS-CoV-2-negative test in that same calendar week in the unvaccinated cohort, to ensure that matched pairs had presence in Qatar over the same time period.

b Severity, criticality, and fatality were defined according to the World Health Organization guidelines.

c Adjusted for sex, 10-year age group, nationality groups, number of coexisting conditions, and calendar week of the second dose in the two-dose cohort or of the SARS-CoV-2-negative test in the unvaccinated cohort.

d Calculated using the adjusted hazard ratio.

e Adjusted for sex, 10-year age group, nationality groups, number of coexisting conditions, calendar week of the second dose in the two-dose cohort or of the SARS-CoV-2-negative test in the unvaccinated cohort, and testing rate.

f Calculated using the adjusted hazard ratio including further adjustment by testing rate.

BNT162b2 effectiveness against symptomatic infection was 71.4% (95% CI: 69.2–73.4) before adjustment for testing rate and increased to 81.2% (95% CI: 79.8–82.5) after adjustment (Table 4 and Fig. 1). BNT162b2 effectiveness against severe, critical, or fatal COVID-19 was 96.8% (95% CI: 94.7–98.1) before adjustment and increased to 98.0% (95% CI: 96.6–98.8) after adjustment.

Vaccine effectiveness of the two-dose primary mRNA-1273 series against any SARS-CoV-2 infection was 67.8% (95% CI: 65.1–70.4) before adjustment for testing rate and increased to 79.4% (95% CI: 77.6–81.0) after adjustment (Table 4 and Fig. 1).mRNA-1273 effectiveness against symptomatic infection was 77.3% (95% CI: 72.8–81.1) before adjustment for testing rate and increased to 84.7% (95% CI: 81.6–87.3) after adjustment (Table 4 and Fig. 1). mRNA-1273 effectiveness against severe, critical, or fatal COVID-19 was 96.6% (95% CI: 89.2–98.9) before adjustment and increased to 97.3% (95% CI: 91.4–99.1) after adjustment.

Effectiveness assessed at 3-month intervals following vaccination showed rapid waning of protection against any and symptomatic infection for both vaccines (Supplementary Figs. S8 and S9, in the supplementary material), whereas protection against severe, critical, or fatal COVID-19 declined only very modestly over time. Without adjustment for testing rate, effectiveness estimates against any and symptomatic infection were negative beyond 6 months, with wide 95% CIs.

#### Additional analysis

The nested analysis, in which the test-negative study was embedded within the cohort study, yielded estimates that were closely aligned with the cohort estimates after adjustment for testing rate (Fig. 3 and Supplementary Table S3, in the supplementary material). This result suggests that the test-negative design is theoretically closely related to the cohort design when differences in testing between vaccinated and unvaccinated individuals are adequately controlled.

## Discussion

Vaccine effectiveness estimates from the test-negative case-control and retrospective cohort designs were similar and consistent across vaccines and outcomes, and aligned with estimates reported in other populations and data sources [6, 33], supporting the validity of both approaches. Both designs also captured the rapid, largely linear waning of effectiveness in magnitude and pattern. However, the findings also corroborate earlier modeling simulations indicating that the cohort design is more susceptible to bias from differential testing by vaccination status [15], highlighting the need to adjust for testing rate in cohort studies to reduce bias and avoid spurious negative effectiveness estimates [34].

This consistency between the two designs is notable because, despite harmonized analytic strategies, they differ subtly in eligibility criteria and in the timing of vaccination and covariate assessment—at the time of testing in the test-negative design and at follow-up initiation in the cohort design [16]. In addition, some differences in the timing of effectiveness measurement relative to the second dose across designs and outcomes, in the context of rapidly waning immunity, would be expected to produce some divergence in estimates. These timing differences reflect the temporal distribution of vaccinations for each of the two vaccines, as well as the temporal distribution of cases (infections and severe outcomes) in the test-negative design during the study period. They arise from variation in vaccine scale-up for the two vaccines (Fig. S6, in the supplementary material), the occurrence and timing of epidemic waves (Fig. S1, in the supplementary material), and differences in severity of variants across waves [35]. Nevertheless, the estimates were closely aligned across both designs despite these differences.

Although overall agreement between designs was strong, systematic differences in estimates were observed, which were largely attributable to differences in the handling of health-seeking behavior, as further supported by the additional test-negative analysis restricted to tests occurring during cohort study follow-up. In analyses without adjustment for testing rate, vaccine effectiveness estimates from the cohort design were typically slightly lower than those from the test-negative design for infection outcomes. However, after adjustment for testing rate, cohort-based estimates increased and exceeded those from the test-negative design.

This pattern reflects differential testing intensity between vaccinated and unvaccinated individuals, which can lead to underascertainment of infections in groups undergoing less frequent testing. By design, the test-negative approach restricts analysis to individuals who undergo testing, thereby inherently partially accounting for differences in testing behavior [7, 8, 15, 36], whereas the cohort design requires explicit adjustment to address this source of bias. However, conditioning on testing in the test-negative design constitutes postbaseline stratification and may introduce selection bias [16]. Together, these findings highlight testing behavior as a key determinant of differences between designs and underscore the importance of accounting for testing intensity in cohort-based analyses. Importantly, such detailed measures of testing behavior may not be readily available in other settings, where testing data are incomplete or not systematically captured, which may limit the ability to address this source of bias and should be considered when interpreting vaccine effectiveness estimates.

A related methodological issue concerns how testing behavior should be measured and incorporated in cohort analyses. In this study, postbaseline testing rate was used because it directly captures testing behavior during follow-up, when outcome ascertainment occurs, and avoids reliance on measures obtained under potentially different testing policies. Notably, a prior vaccine effectiveness analysis in the same population using a cohort design has demonstrated similar results when adjusting for either prebaseline or postbaseline testing rates [37].

Taken together, these findings do not indicate that either design is inherently superior. Rather, they emphasize that credible vaccine effectiveness estimation hinges on rigorous implementation, including stringent eligibility criteria, thorough measurement of potential confounders, and close matching or adjustment for factors related to both vaccination and infection risk. When feasible, applying both designs within the same population can enhance confidence in estimates and help identify and address design-specific sources of bias or confounding.

Lastly, while modest differences across designs were observed for effectiveness against any or symptomatic infection, estimates for protection against severe, critical, or fatal COVID-19 were virtually identical across designs. This may reflect the limited influence of testing behavior on severe outcomes and the very high level of protection against severe disease, as the impact of bias or confounding is typically smaller at higher levels of effectiveness [15, 16].

This study has limitations. The observed consistency between the test-negative and cohort designs was demonstrated in a specific national setting with a predominantly young, relatively healthy population and may not generalize to other populations, time periods, or infections. As with all observational studies, bias cannot be excluded and may arise from unmeasured or imperfectly measured factors [9, 38]. Misclassification of exposures, outcomes, or covariates is also possible and, although likely non-differential, could attenuate or distort effect estimates.

Matching reduced confounding but may not have eliminated it entirely and could have introduced selection bias [39]. Matching was not performed on geographic location or occupation; however, the use of age, sex, and nationality as proxies for socioeconomic status in this setting may have partially mitigated residual confounding [32, 40–43]. This approach has been supported by prior studies in the same population, in which similar matching strategies were validated using negative control analyses [9, 18, 44–46]. Further discussion of limitations specific to each design is provided in Supplementary Section 6 (test-negative design), in the supplementary materials online, and Supplementary Section 7 (cohort design), in the supplementary materials online.

This study has strengths. Effectiveness was evaluated across two mRNA vaccines and three clinically relevant outcomes using very large, nationally representative datasets, enabling high statistical precision. The use of comprehensive, validated national databases, and an integrated digital health platform allowed detailed capture of covariates and rigorous matching [21, 47, 48].

In conclusion, vaccine effectiveness estimates derived from the test-negative case-control and retrospective cohort designs were consistent across different vaccines and clinically relevant outcomes. These findings provide empirical evidence that, when rigorously implemented using quality data and harmonized analytic strategies, both designs can generate comparable effectiveness estimates. Rather than favoring one design over the other, the results underscore the importance of rigorous implementation, careful handling of confounding—particularly health-seeking behavior—and, when feasible, triangulation across complementary study designs to strengthen inference and confidence in real-world effectiveness estimates.

## Ethics approval

The institutional review boards at Hamad Medical Corporation and Weill Cornell Medicine-Qatar approved this retrospective study with a waiver of informed consent. The study was reported according to the Strengthening the Reporting of Observational Studies in Epidemiology (STROBE) guidelines for case-control and cohort studies (Supplementary Tables S4 and S5, in the supplementary material, respectively).

## Supplementary Material

dyag210_Supplementary_Data

## Data Availability

The dataset of this study is a property of the Qatar Ministry of Public Health that was provided to the researchers through a restricted-access agreement that prevents sharing the dataset with a third party or publicly. The data are available under restricted access for preservation of confidentiality of patient data. Access can be obtained through a direct application for data access to His Excellency the Minister of Public Health (https://emsfsa.moph.gov.qa/en/Pages/eservices.aspx). The raw data are protected and are not available due to data privacy laws. Requests for access are assessed by the Ministry of Public Health in Qatar, and approval is granted at its discretion. In compliance with data privacy laws and the data-sharing agreement with the Ministry of Public Health in Qatar, no datasets, whether raw or de-identified, can be publicly released by the researchers. Aggregate data are available within the paper and its supplementary information.
