# Supplementary material for: Evaluating COVID-19 vaccine effectiveness across epidemiologic designs: test-negative case-control versus retrospective cohort analyses in a nationwide study
Source: Int J Epidemiol. 2026 Sep 22;55(5):dyag210. doi: 10.1093/ije/dyag210 (PMC13597121; doi:10.1093/ije/dyag210)
Supplement: Supplementary file 1 [file dyag210_supplementary_data.docx]

**Supplementary Material**

**Table of Contents**

[**Section 1. Background and rationale for study design comparison**	3](#_Toc238555607)

[**Section 2. Study population and data sources**	6](#_Toc238555608)

[**Section 3. Laboratory methods and variant ascertainment**	9](#_Toc238555609)

[**Real-time reverse-transcription polymerase chain reaction testing**	9](#_Toc238555610)

[**Classification of infections by variant type**	9](#_Toc238555611)

[**Section 4. Classification of coexisting conditions**	10](#_Toc238555612)

[**Figure S1. Daily counts of newly diagnosed SARS-CoV-2 infections during the study period, from 1 January 2021 to 18 December 2021.**	12](#_Toc238555613)

[**Section 5. COVID-19 severity, criticality, and fatality classification**	13](#_Toc238555614)

[**Severe COVID-19**	13](#_Toc238555615)

[**Critical COVID-19**	13](#_Toc238555616)

[**Fatal COVID-19**	14](#_Toc238555617)

[**Section 6. Test-negative case-control study**	15](#_Toc238555618)

[**Limitations and strengths**	16](#_Toc238555619)

[**Section 7. Retrospective cohort study**	18](#_Toc238555620)

[**Limitations and strengths**	20](#_Toc238555621)

[**Figure S2. Population selection process in the test-negative case-control study evaluating BNT162b2 primary-series vaccine effectiveness against SARS-CoV-2 infection.**	22](#_Toc238555622)

[**Figure S3. Population selection process in the test-negative case-control study evaluating mRNA-1273 primary-series vaccine effectiveness against SARS-CoV-2 infection.**	23](#_Toc238555623)

[**Figure S4. Population selection process in the retrospective cohort study evaluating BNT162b2 primary-series vaccine effectiveness against SARS-CoV-2 infection.**	24](#_Toc238555624)

[**Figure S5. Population selection process in the retrospective cohort study evaluating mRNA-1273 primary-series vaccine effectiveness against SARS-CoV-2 infection.**	25](#_Toc238555625)

[**Section 8. Statistical analysis**	26](#_Toc238555626)

[**Test-negative case-control study**	26](#_Toc238555627)

[**Retrospective cohort study**	26](#_Toc238555628)

[**Figure S6. Daily counts of second-dose vaccinations for A) BNT162b2 and B) mRNA-1273 during the study period, from 1 January 2021 to 18 December 2021.**	29](#_Toc238555629)

[**Table S1. Characteristics of the eligible unmatched cases and controls in the BNT162b2 and mRNA-1273 analyses of the test-negative case-control study.**	30](#_Toc238555630)

[**Table S2. Baseline characteristics of the eligible unmatched vaccinated and unvaccinated cohorts in the BNT162b2 and mRNA-1273 analyses of the retrospective cohort study.**	31](#_Toc238555631)

[**Figure S7. Vaccine effectiveness at 3-month intervals following primary series vaccination with A) BNT162b2 and B) mRNA-1273 in the test-negative case-control study. Effectiveness is shown against any SARS-CoV-2 infection, symptomatic infection, and severe, critical, or fatal COVID-19.**	32](#_Toc238555632)

[**Figure S8. Vaccine effectiveness at 3-month intervals following primary series vaccination with BNT162b2 in the retrospective cohort study A) without adjustment for testing rate and B) with adjustment for testing rate. Effectiveness is shown against any SARS-CoV-2 infection, symptomatic infection, and severe, critical, or fatal COVID-19.**	33](#_Toc238555633)

[**Figure S9. Vaccine effectiveness at 3-month intervals following primary series vaccination with mRNA-1273 in the retrospective cohort study A) without adjustment for testing rate and B) with adjustment for testing rate. Effectiveness is shown against any SARS-CoV-2 infection, symptomatic infection, and severe, critical, or fatal COVID-19.**	34](#_Toc238555634)

[**Table S3. Additional analysis restricting the test-negative design to SARS-CoV-2-positive and -negative tests occurring during cohort study follow-up for primary-series BNT162b2 and mRNA-1273 vaccination, against any SARS-CoV-2 infection, symptomatic infection, and severe, critical, or fatal COVID-19.**	35](#_Toc238555635)

[**Table S4. Strengthening the Reporting of Observational Studies in Epidemiology (STROBE) checklist for case-control studies.**	36](#_Toc238555636)

[**Table S5. Strengthening the Reporting of Observational Studies in Epidemiology (STROBE) checklist for cohort studies.**	38](#_Toc238555637)

[**References**	40](#_Toc238555638)

# **Section 1. Background and rationale for study design comparison**

While randomized controlled trials (RCTs) offer high internal validity and remain the gold standard for establishing vaccine efficacy, their external validity is often constrained by selective enrollment, underrepresentation of individuals with coexisting conditions, controlled study conditions, relatively short follow-up durations, and a limited range of assessed clinical outcomes^1-3^. As a result, RCTs may not reflect vaccine performance under routine clinical practice and population-level implementation^1, 2^.

Real-world observational studies therefore play a central role in assessing vaccine effectiveness beyond the trial setting and have been instrumental in informing public health policies and vaccination programs, such as during the coronavirus disease (COVID-19) pandemic^4-6^. Two observational designs are most commonly used for this purpose: the retrospective cohort design^6, 7^, often implemented using a target trial–inspired framework^8, 9^, and the test-negative case-control design^6, 10-12^.

In real-world settings, heterogeneity in health status, healthcare access, health-seeking behavior, and broader structural determinants can influence both vaccine uptake and outcome ascertainment, thereby potentially distorting effectiveness estimates^5, 13-15^. Effectiveness estimates derived from observational studies may therefore be affected by multiple sources of bias, including confounding, selection bias, and information (misclassification) bias, as well as residual bias arising from incomplete or imperfect measurement of relevant factors^5, 13, 15, 16^. Confounding arises when factors related to both vaccination and risk of infection or disease are not fully accounted for, while selection and information biases may be introduced through differential inclusion, testing, or outcome ascertainment^5, 13, 15, 16^. The magnitude and direction of these biases cannot be assumed a priori and depend on the specific study context, including patterns of testing, data completeness, measurement of key variables, and analytic choices, rather than on study design alone^5, 13, 15, 16^.

A key strength of the cohort design, particularly when implemented to emulate a target trial, is its capacity to reduce design-induced bias through explicit alignment of eligibility criteria, exposure definition, follow-up, and outcome assessment^8, 9, 16, 17^. However, achieving a fully faithful target trial in practice is challenging, and deviations from the ideal framework can reintroduce bias, with health-seeking behavior being a particularly influential factor that may differentially affect exposure classification and outcome detection^16^.

The test-negative design offers an important advantage in partially controlling for health-seeking behavior by restricting analyses to individuals who seek care and undergo testing^10, 11, 15, 18^. However, this restriction may itself introduce design-induced bias, as conditioning on testing constitutes postbaseline selection and can induce selection bias if testing is influenced by vaccination status or other factors related to infection risk^10, 15, 16, 18^. Thus, although each design addresses certain sources of bias, each is also susceptible to distinct limitations^16^.

Despite the widespread application of both designs across a range of infections, direct empirical comparisons of their real-world performance—applied to the same population, during the same time period, and using harmonized analytic approaches—remain limited^16^. In this study, the real-world application and performance of the test-negative case-control and retrospective cohort designs were evaluated within a single national population, using closely aligned analytic strategies. This approach enabled a direct assessment of the extent to which the two designs yield consistent estimates of vaccine effectiveness and facilitated exploration of potential reasons for any observed differences.

The analysis leveraged comprehensive national-level databases in Qatar, enabling the implementation of both designs with large, population-based sample sizes. Comparisons were conducted separately for two mRNA vaccines and evaluated protection against three clinically relevant outcomes—any severe acute respiratory syndrome coronavirus 2 (SARS-CoV-2) infection, symptomatic infection, and severe, critical, or fatal COVID-19. This framework allowed a rigorous assessment of design performance across distinct clinical endpoints and epidemiologic contexts.

# **Section 2. Study population and data sources**

This study was conducted in the population of Qatar between 1 January 2021, following the launch of the COVID-19 primary series vaccination program^19, 20^, and 18 December 2021, the day preceding the onset of the first Omicron wave^21^. The analysis was deliberately restricted to the pre-Omicron period, when vaccine effectiveness was high owing to closer antigenic concordance between vaccine composition and circulating SARS-CoV-2 variants^6^, and when estimates were less susceptible to potential confounding factors arising in later phases of the pandemic^22-26^.

The vaccine roll-out coincided with the introduction of the Alpha variant in Qatar^27^, which was then replaced by the Beta variant, the most immune-evasive variant circulating in the country before Omicron^28^. This was followed by a prolonged phase of low incidence dominated by the Delta variant^29^ (Supplementary Figure S1).

Data were obtained from Qatar’s integrated national digital health platform, which captures all COVID-19 laboratory testing, vaccinations, hospitalizations, and deaths nationwide. The database provides complete national coverage of polymerase chain reaction (PCR) testing from the start of the pandemic, irrespective of testing site (Supplementary Section S3). During the study period, approximately 5% of Qatar’s population underwent weekly PCR testing, largely for routine screening, leading to the identification of most infections preemptively rather than through symptom-driven testing^12, 30^.

Qatar’s vaccination campaign began at the end of December 2020 with BNT162b2, followed three months later by mRNA-1273^31-33^. Vaccination was tracked nationally, offered free of charge to all individuals, and initially prioritized for older adults and individuals with coexisting health conditions^12, 33^.

Demographic information, including sex, age, and nationality, was obtained from the national health registry. Qatar’s population is heterogeneous, with expatriates accounting for 89% of residents and representing more than 150 nationalities^34^. Detailed descriptions of the study population and data sources have been reported previously^7, 12, 23, 30, 34, 35^.

Qatar’s national and universal public healthcare system uses the Cerner Millennium electronic medical record (EMR) system to track all public healthcare encounters of each individual in the country, including all citizens and residents registered in the national and universal public healthcare system. Registration in the public healthcare system is mandatory for citizens and residents.

The databases analyzed in this study are data-extract downloads from the national EMR database that have been implemented on a regular weekly schedule since the onset of the pandemic by the Business Intelligence Unit at Hamad Medical Corporation (HMC). HMC is the national public healthcare provider in Qatar. At every download, all SARS-CoV-2 tests, COVID-19 vaccinations, hospitalizations related to COVID-19, and all death records regardless of cause are provided to the authors through .csv files. These databases have been analyzed throughout the pandemic not only for study-related purposes, but also to provide policymakers with summary data and analytics to inform the national response.

Every health encounter in the national EMR is linked to an individual through the HMC Number, which serves as a unique identifier that links all records for this individual at the national level. Databases were merged and analyzed using the HMC Number to link all records pertaining to testing, vaccinations, hospitalizations, and deaths. All deaths in Qatar are recorded by the public healthcare system. COVID-19-related healthcare was provided exclusively in the public healthcare system. COVID-19 vaccination was also provided only through the public healthcare system. These health records were tracked throughout the COVID-19 pandemic using the national EMR system. This system has been implemented in 2013, before the onset of the pandemic. This pre-established system ensured that we had access to comprehensive health records related to this study for both citizens and residents throughout the pandemic, allowing us to follow each person over time.

SARS-CoV-2 testing in any facility in Qatar is tracked nationally in one database, the national testing database. This database covers all testing throughout the country, whether in public or private facilities. Every PCR test conducted in Qatar, regardless of location or setting, is classified on the basis of symptoms and the reason for testing, such as the presence of clinical symptoms, contact tracing, participation in surveys or random testing campaigns, individual requests for testing, routine healthcare testing, pre-travel requirements, at the point of entry into the country, or any other relevant reasons for testing.

# **Section 3. Laboratory methods and variant ascertainment**

## **Real-time reverse-transcription polymerase chain reaction testing**

Nasopharyngeal and/or oropharyngeal swabs were collected for PCR testing and placed in Universal Transport Medium (UTM). Aliquots of UTM were: 1) extracted on KingFisher Flex (Thermo Fisher Scientific, USA), MGISP-960 (MGI, China), or ExiPrep 96 Lite (Bioneer, South Korea) followed by testing with real-time reverse-transcription PCR (RT-qPCR) using TaqPath COVID-19 Combo Kits (Thermo Fisher Scientific, USA) on an ABI 7500 FAST (Thermo Fisher Scientific, USA); 2) tested directly on the Cepheid GeneXpert system using the Xpert Xpress SARS-CoV-2 (Cepheid, USA); or 3) loaded directly into a Roche cobas 6800 system and assayed with the cobas SARS-CoV-2 Test (Roche, Switzerland). The first assay targets the viral S, N, and ORF1ab gene regions. The second targets the viral N and E-gene regions, and the third targets the ORF1ab and E-gene regions.

All PCR testing was conducted at the HMC Central Laboratory or Sidra Medicine Laboratory, following standardized protocols.

## **Classification of infections by variant type**

Surveillance for SARS-CoV-2 variants in Qatar is based on viral genome sequencing and multiplex RT-qPCR variant screening^36^ of weekly collected random positive clinical samples^12, 19, 20, 37-39^, complemented by deep sequencing of wastewater samples^37, 40, 41^. Further details on the viral genome sequencing and multiplex RT-qPCR variant screening throughout the SARS-CoV-2 waves in Qatar can be found in previous publications^7, 12, 19-21, 29, 30, 37-39, 42-46^.

# **Section 4. Classification of coexisting conditions**

The classification of patients and their coexisting conditions was carried out by an independent medical team appointed by the Ministry of Public Health. This process was part of routine needs assessment exercises aimed at planning healthcare use, utilization, and resource allocation.

As Qatar’s medical coding systems evolved over time, incorporating different versions of ICD codes as well as SNOMED codes, the medical team developed mappings to harmonize these codes and ensure consistent categorization of coexisting conditions. The study investigators did not have direct access to the detailed procedures, documentation, or specific codes and mappings used by this independent team. Instead, the finalized database of patients and their coexisting conditions was provided to the study investigators exclusively for research purposes.

Coexisting conditions were ascertained and classified based on the mapped codes for the conditions, as recorded in the electronic health record encounters of each individual in the national EMR database that includes all citizens and residents registered in the national and universal public healthcare system. The public healthcare system provides healthcare to the entire resident population of Qatar free of charge or at heavily subsidized costs, including prescription drugs. With the mass expansion of this sector in recent years, facilities have been built to cater to specific needs of subpopulations. For example, tens of facilities have been built, including clinics and hospitals, in localities with high density of craft and manual workers^47^.

All encounters for each individual were analyzed to determine the coexisting-condition classification for that individual. The national EMR database includes encounters starting from 2013, when this system was launched in Qatar. Any individual who had at least one encounter with a specific coexisting-condition diagnosis since 2013 was classified as having that coexisting condition. Individuals who do not have records of coexisting-condition encounters in the public healthcare system were classified as having no coexisting conditions.

The classification of coexisting conditions spanned the following conditions: 1) Behchet’s disease, 2) cancer, 3) cardiovascular diseases, 4) infectious and parasitic diseases, 5) Crohn’s disease, 6) chronic kidney disease (CKD), 7) chronic liver disease (CLD), 8) chronic lung disease, 9) congenital malformations, deformations and chromosomal abnormalities, 10) diseases of the blood and blood-forming organs and certain disorders involving the immune mechanism, 11) diseases of the ear and mastoid process, 12) deep vein thrombosis (DVT), 13) dermatitis, 14) diabetes mellitus, 15) diseases of the circulatory system, 16) diseases of the digestive system, 17) diseases of the eye and adnexa, 18) diseases of the genitourinary system, 19) diseases of the musculoskeletal system and connective tissue, 20) diseases of the nervous system, 21) diseases of the respiratory system, 22) diseases of the skin and subcutaneous tissue, 23) endocrine, nutritional and metabolic diseases, 24) gingivitis, 25) hypertension, 26) injury, poisoning and certain other consequences of external causes, 27) mental and behavioral disorders, 28) neoplasms, 29) periodontitis, 30) pregnancy, childbirth and the puerperium, 31) pulmonary tuberculosis, 32) rheumatoid arthritis, 33) Sjogren’s syndrome, 34) stroke or neural conditions, 35) symptoms, signs and abnormal clinical and laboratory findings, not elsewhere classified, 36) systemic lupus erythematosus, 37) systemic sclerosis, 38) organ transplant, and 39) other unspecified factors influencing health status and contact with health services.

# **Figure S1.** **Daily counts of newly diagnosed SARS-CoV-2 infections during the study period, from 1 January 2021 to 18 December 2021.**

SARS-CoV-2 denotes severe acute respiratory syndrome coronavirus 2.

Alt text: Epidemic curve shows daily SARS-CoV-2 infections rising during Alpha and Beta waves early in 2021, then declining to a prolonged lower-incidence period dominated by Delta.

# **Section 5. COVID-19 severity, criticality, and fatality classification**

Classification of COVID-19 case severity (acute-care hospitalizations)^48^, criticality (intensive-care-unit hospitalizations)^48^, and fatality^49^ followed World Health Organization (WHO) guidelines. Assessments were made by trained medical personnel independent of study investigators and using individual chart reviews, as part of a national protocol applied to every hospitalized COVID-19 patient. Each hospitalized COVID-19 patient underwent an infection severity assessment every three days until discharge or death. We classified individuals who progressed to severe, critical, or fatal COVID-19 between the time of the documented infection and the end of the study based on their worst outcome, starting with death^49^, followed by critical disease^48^, and then severe disease^48^.

## **Severe COVID-19**

Severe COVID-19 disease was defined per WHO classification as a SARS-CoV-2-infected person with “oxygen saturation of <90% on room air, and/or respiratory rate of >30 breaths/minute in adults and children >5 years old (or ≥60 breaths/minute in children <2 months old or ≥50 breaths/minute in children 2-11 months old or ≥40 breaths/minute in children 1–5 years old), and/or signs of severe respiratory distress (accessory muscle use and inability to complete full sentences, and, in children, very severe chest wall indrawing, grunting, central cyanosis, or presence of any other general danger signs)”^48^. Detailed WHO criteria for classifying SARS-CoV-2 infection severity can be found in the WHO technical report^48^.

## **Critical COVID-19**

Critical COVID-19 disease was defined per WHO classification as a SARS-CoV-2-infected person with “acute respiratory distress syndrome, sepsis, septic shock, or other conditions that would normally require the provision of life-sustaining therapies such as mechanical ventilation (invasive or non-invasive) or vasopressor therapy”^48^. Detailed WHO criteria for classifying SARS-CoV-2 infection criticality can be found in the WHO technical report^48^.

## **Fatal COVID-19**

COVID-19 death was defined per WHO classification as “a death resulting from a clinically compatible illness, in a probable or confirmed COVID-19 case, unless there is a clear alternative cause of death that cannot be related to COVID-19 disease (e.g. trauma). There should be no period of complete recovery from COVID-19 between illness and death. A death due to COVID-19 may not be attributed to another disease (e.g. cancer) and should be counted independently of preexisting conditions that are suspected of triggering a severe course of COVID-19”^49^. Detailed WHO criteria for classifying COVID-19 death can be found in the WHO technical report^49^.

# **Section 6. Test-negative case-control study**

Effectiveness was estimated by comparing the odds of two-dose vaccination among cases (SARS-CoV-2-positive tests) versus controls (SARS-CoV-2-negative tests)^10-12, 50^. Effectiveness was defined as the proportional reduction in susceptibility to infection among vaccinated versus unvaccinated individuals (reference group)^10-12, 50, 51^.

SARS-CoV-2 tests conducted in Qatar during the study period were classified according to symptom status and reason for testing. The outcome of *any SARS-CoV-2 infection* was defined as a SARS-CoV-2-positive test irrespective of testing indication, whereas *symptomatic SARS-CoV-2 infection* was defined as a SARS-CoV-2-positive test performed because of symptoms consistent with a respiratory tract infection.

COVID-19 severity was classified according to World Health Organization (WHO) criteria for severe disease (acute-care hospitalization)^48^, critical disease (intensive-care unit hospitalization)^48^, and fatal disease^49^. Severity determinations were performed independently by trained medical personnel through systematic chart reviews^52, 53^ (Supplementary Section S5). Under national protocol, all hospitalized SARS-CoV-2-positive patients underwent clinical assessment every three days until discharge or death^52, 53^. Each patient was assigned a single outcome corresponding to the maximum severity reached, with outcomes hierarchically ordered as fatal, followed by critical, and then severe disease.

Only the first SARS-CoV-2-positive test per individual during the study period was included, whereas all SARS-CoV-2-negative tests were eligible for inclusion^50^. Individuals were excluded if, before the index test, they had evidence of prior infection, had received an incomplete primary series (one dose only), had received three or more doses, had been vaccinated with non-mRNA vaccines, had received mixed vaccination regimens, or had been tested within 14 days after receipt of the second dose^50^.

These inclusion and exclusion criteria allowed for the build-up of vaccine immunity post-vaccination^31, 32^ and minimized potential biases identified in previous analyses of the same population^12, 50, 54, 55^. All eligible cases and controls that could be matched were included in the analysis.

Cases and controls were exactly matched one-to-one for any or symptomatic SARS-CoV-2 infection, but one-to-five for severe COVID-19, to enhance statistical precision given the smaller number of severe outcomes. Exact matching was performed on sex, 10-year age group, nationality, number of coexisting conditions (0 to ≥6; Supplementary Section S4), calendar week of the SARS-CoV-2 test, and reason for testing.

The matching factors comprised potential confounders associated with both the likelihood of vaccination and the risk of infection outcomes^16^. By design, exact matching ensured identical distributions of these characteristics between cases and controls, thereby controlling for confounding related to differential vaccination uptake or infection risk across comparison groups^34, 47, 56-58^. Matching variables were selected a priori informed by prior COVID-19 epidemiologic studies conducted in Qatar’s population^12, 20, 33, 54^.

## **Limitations and strengths**

This analysis has limitations. Bias inherent to real-world data cannot be excluded, including residual differences in health-seeking and testing behavior^12, 55^. Although the test-negative design conditions on testing, which improves comparability between cases and controls, this conditioning may not fully eliminate differences in testing patterns by vaccination status or infection risk.

Some infections may have gone undetected. However, SARS-CoV-2 testing in Qatar was extensive and largely routine^12, 30^, making differential underascertainment within the tested population unlikely to have materially biased the study findings. Symptomatic infection was defined by the documented reason for testing. Some very mild or incompletely documented symptomatic infections may therefore have been missed, but this definition was applied consistently to cases and controls.

The test-negative design may also be affected by potential bias if vaccination altered the clinical presentation of infection, thereby influencing the likelihood of symptoms prompting testing or the probability of infection detection. In such situations, vaccinated individuals with milder or asymptomatic infections may be less likely to undergo symptom-driven testing, which could lead to overestimation of effectiveness against symptomatic infection. In addition, because only the first SARS-CoV-2-positive test per individual was included, the analysis did not account for recurrent infections during the study period, which may have influenced the estimation of protection in populations with heterogeneous exposure risk.

This analysis has strengths. Cases and controls were drawn from the same national testing system and were exactly matched on key factors, including calendar week and testing indication. This controlled for differences in epidemic phase, variant circulation, changing testing policies, and reason for testing. Conditioning on testing also accounted for health-seeking and testing behavior^10^.

# **Section 7. Retrospective cohort study**

This study compared the incidence of infection between individuals who received two vaccine doses (two-dose cohort) and those who were unvaccinated (unvaccinated cohort), building on cohort designs previously developed and applied in the same population^7, 23, 24, 59, 60^. Effectiveness was estimated by comparing the incidence of SARS-CoV-2 infection and of severe COVID-19 between these cohorts.

Incidence of any SARS-CoV-2 infection was defined as the first SARS-CoV-2-positive test occurring after initiation of follow-up, irrespective of symptom status. Incidence of symptomatic infection was defined as the first SARS-CoV-2-positive test after follow-up initiation that was conducted due to the presence of clinical symptoms. Severe COVID-19 outcomes were classified according to the maximum severity attained and were assigned to the date of the SARS-CoV-2-positive test confirming infection.

Individuals in the two-dose cohort were exactly matched one-to-one with individuals in the unvaccinated cohort on sex, 10-year age group, nationality, and number of coexisting conditions (0 to ≥6; Supplementary Section S4). This exact matching ensured identical distributions of these characteristics between cohorts and controlled for observable confounders influencing infection risk across exposure groups^34, 47, 56-58^, consistent with the approach used in the test-negative study.

Individuals in the two-dose cohort who received their second dose in a given calendar week were matched to individuals in the unvaccinated cohort with a SARS-CoV-2-negative test recorded in the same calendar week^14, 23^. This approach ensured comparability at cohort entry by aligning calendar time and exposure to vaccination policies and public health measures. The requirement for a same-week negative test among unvaccinated individuals was not intended to condition on a negative outcome, but rather to serve as a baseline eligibility and time-anchoring criterion. Because vaccinated individuals had a documented vaccination encounter confirming presence in Qatar at cohort entry, the same-week negative test provided corresponding objective evidence that unvaccinated individuals were present in Qatar, at risk, and subject to the same epidemic conditions^14, 23^.

In the absence of a complete population registry with real-time residency status, and given that mainly COVID-19–related healthcare encounter data were available, this approach provided a practical means to confirm contemporaneous presence in the population. Although alternative indicators of presence could be considered, the national SARS-CoV-2 testing database represented the most complete and consistently time-stamped source for this purpose. This approach is further supported by the context of the COVID-19 pandemic in Qatar, during which testing was extensive and frequently mandated for travel, occupational screening, and other public health requirements. Consequently, the number of individuals tested during the study period was nearly equivalent to the total population size, indicating that the tested population closely approximates the population under observation. Taking these considerations into account, this approach is not likely to have introduced bias in the estimated vaccine effectiveness or to have affected the validity of the study conclusions.

Each matched pair was followed from 14 days after receipt of the second dose to allow adequate time for post-vaccination immune development^31, 32^. Eligibility for the two-dose cohort required completion of a two-dose mRNA vaccination series. Individuals were excluded if they had received a non-mRNA vaccine, had mixed vaccination regimens, died before follow-up initiation, had not completed 14 days after the second dose by the end of the study period, or had a discrepant vaccination schedule. In the unvaccinated cohort, individuals with SARS-CoV-2 tests recorded after death were excluded. Participants in both cohorts were excluded if they had evidence of prior SARS-CoV-2 infection or if death dates were unascertained or inconsistent.

Matching was performed using an iterative algorithm^14, 23^ that selected individuals from the reference (unvaccinated) cohort, ensuring that, at follow-up initiation, they were alive, had no vaccination record, and had no documented prior SARS-CoV-2 infection. The algorithm was implemented in Stata using the *ccmatch* command, with additional eligibility constraints applied to ensure retention of valid controls. The matching procedure was repeated iteratively until exhaustion, defined as the point at which no additional matched pairs could be identified.

Under this study design, individuals in the unvaccinated cohort could contribute person-time before receipt of their primary series and subsequently contribute additional person-time as members of the two-dose cohort after vaccination.

To preserve exchangeability^7, 61^, both members of each matched pair were censored at the earliest occurrence of receipt of a new vaccine dose. Accordingly, individuals were followed until the first occurrence of documented SARS-CoV-2 infection, receipt of a first vaccine dose in the unvaccinated cohort (with matched-pair censoring), receipt of a third vaccine dose in the two-dose cohort (with matched-pair censoring), death, or administrative censoring at the end of the study period.

## **Limitations and strengths**

This analysis has limitations. Unvaccinated individuals were identified using the national testing database, which does not capture persons who were never tested. Nevertheless, SARS-CoV-2 testing in Qatar was extensive and largely routine^12, 30^, making it unlikely that substantial numbers of residents remained untested. Although some infections may have gone undetected, there is no evident reason to expect differential underascertainment between cohorts that would materially bias the study findings.

The retrospective cohort design may also be susceptible to bias arising from differential depletion of susceptible individuals over time, particularly during periods of high infection incidence^62, 63^. Because unvaccinated individuals are generally at higher risk of infection earlier during follow-up, the remaining unvaccinated cohort may progressively become enriched with individuals at lower underlying risk or with undocumented prior immunity, potentially attenuating effectiveness estimates at later time points^62, 63^. In addition, although adjustment for testing rate was implemented, testing intensity may vary over time and may not be fully captured by broad testing-rate categories, leaving the possibility of residual differential ascertainment of infection outcomes. Furthermore, proportional hazards assumptions were not fully satisfied, reflecting expected time-varying vaccine effects due to waning immunity. Consequently, hazard ratios should be interpreted as average effects over follow-up rather than constant effects over time.

This analysis has strengths. The cohort design explicitly emulated key elements of a target trial by defining eligibility, exposure, time zero, follow-up, censoring, and outcome ascertainment before analysis, thereby reducing time-related and immortal-time biases. The same-week matching criterion provided objective evidence that unvaccinated comparators were present in Qatar, at risk, and under observation during the same epidemic period as the matched vaccinated individuals. Matched-pair censoring when vaccination status changed further helped preserve comparability between cohorts during follow-up. Adjustment for testing rate was also implemented to reduce bias arising from differential testing intensity by vaccination status during follow-up.

# **Figure S2. Population selection process in the test-negative case-control study evaluating BNT162b2 primary-series vaccine effectiveness against SARS-CoV-2 infection.**

SARS-CoV-2 denotes severe acute respiratory syndrome coronavirus 2.

^*^SARS-CoV-2-positive tests were matched exactly one-to-one to SARS-CoV-2-negative tests by sex, 10-year age group, nationality, number of coexisting conditions, calendar week of test, and reason for testing.

Alt text: Flowchart tracing BNT162b2 test-negative study eligibility through sequential exclusions, resulting in matched samples of 200,541 SARS-CoV-2-positive tests and 200,541 SARS-CoV-2-negative tests.

# **Figure S3. Population selection process in the test-negative case-control study evaluating mRNA-1273 primary-series vaccine effectiveness against SARS-CoV-2 infection.**

SARS-CoV-2 denotes severe acute respiratory syndrome coronavirus 2.

^*^SARS-CoV-2-positive tests were matched exactly one-to-one to SARS-CoV-2-negative tests by sex, 10-year age group, nationality, number of coexisting conditions, calendar week of test, and reason for testing.

Alt text: Flowchart tracing mRNA-1273 test-negative study eligibility through sequential exclusions, resulting in matched samples of 186,968 SARS-CoV-2-positive tests and 186,968 SARS-CoV-2-negative tests.

# **Figure S4. Population selection process in the retrospective cohort study evaluating BNT162b2 primary-series vaccine effectiveness against SARS-CoV-2 infection.**

COVID-19 denotes coronavirus disease and SARS-CoV-2, severe acute respiratory syndrome coronavirus 2.

^*^Cohorts were matched exactly one-to-one by sex, 10-year age group, nationality, and number of coexisting conditions. Individuals who received their second dose in a specific calendar week in the two-dose cohort were additionally matched to individuals who had a record for a SARS-CoV-2-negative test in that same calendar week in the unvaccinated cohort, to ensure that matched pairs had presence in Qatar over the same time period. Individuals in the unvaccinated cohort had to be alive, with no previous infection preceding the start of follow-up, and with no vaccination record at the start of the follow-up. Each matched pair was followed from 14 days after the date of the second vaccine dose for the individual in the two-dose cohort.

Alt text: Flowchart tracing BNT162b2 cohort eligibility and their unvaccinated comparators, resulting in matched samples of 536,788 vaccinated and 536,788 unvaccinated individuals.

# **Figure S5. Population selection process in the retrospective cohort study evaluating mRNA-1273 primary-series vaccine effectiveness against SARS-CoV-2 infection.**

COVID-19 denotes coronavirus disease and SARS-CoV-2, severe acute respiratory syndrome coronavirus 2.

^*^Cohorts were matched exactly one-to-one by sex, 10-year age group, nationality, and number of coexisting conditions. Individuals who received their second dose in a specific calendar week in the two-dose cohort were additionally matched to individuals who had a record for a SARS-CoV-2-negative test in that same calendar week in the unvaccinated cohort, to ensure that matched pairs had presence in Qatar over the same time period. Individuals in the unvaccinated cohort had to be alive, with no previous infection preceding the start of follow-up, and with no vaccination record at the start of the follow-up. Each matched pair was followed from 14 days after the date of the second vaccine dose for the individual in the two-dose cohort.

Alt text: Flowchart tracing mRNA-1273 cohort eligibility and their unvaccinated comparators, resulting in matched samples of 344,797 vaccinated and 344,797 unvaccinated individuals.

# **Section 8. Statistical analysis**

Statistical analyses were conducted in STATA/SE version 19.0 (Stata Corporation, College Station, TX, USA). Groups or cohorts were described using frequency distributions and measures of central tendency and compared using standardized mean differences (SMDs)^64^. An SMD of ≤0.1 indicated adequate matching^64^.

## **Test-negative case-control study**

All SARS-CoV-2 testing records in this study were reviewed for the selection of cases and controls, but only matched samples were included in the analysis. The median and interquartile range (IQR) of the duration between vaccination and study SARS-CoV-2 test were calculated.

Odds ratios (ORs), comparing the odds of receiving primary series vaccination among cases versus controls, were estimated using conditional logistic regression, along with corresponding 95% confidence intervals (CIs). CIs were not adjusted for multiplicity and interactions were not assessed.

Vaccine effectiveness and corresponding 95% CI were calculated as 1–OR when the OR was <1, and as 1/OR–1 when the OR was ≥1^10, 23, 65^, producing a symmetric scale from –100% to 100% for intuitive interpretation of both positive and negative effectiveness^23, 65^. Effectiveness over time was further assessed using 3-month intervals following vaccination.

## **Retrospective cohort study**

The cumulative incidence of infection (or severe COVID-19), defined as the proportion of individuals at risk whose primary endpoint during follow-up was an infection, was estimated using the Kaplan-Meier method.

Incidence rate of infection in each cohort, defined as the number of identified infections divided by the number of person-weeks contributed by all individuals in the cohort, was estimated along with the corresponding 95% CI using a Poisson log-likelihood regression model with the Stata *stptime* command.

Overall adjusted hazard ratios (aHRs) with corresponding 95% CIs were estimated using Cox regression models to compare the incidence of infection between cohorts. Analyses were conducted in Stata using the *stcox* command, with adjustment for matching factors to ensure robust variance estimation^66^. CIs were not adjusted for multiplicity, and interaction effects were not examined.

To account for potential differences in testing frequency between cohorts, additional analyses were conducted using the same methods, with overall hazard ratios further adjusted for testing rate. Individuals were classified according to their testing intensity during the study period as low testers (0–1 test per person-year), intermediate testers (>1–3 tests per person-year), or high testers (>3 tests per person-year). This adjustment was warranted because most SARS-CoV-2 testing in Qatar was performed routinely rather than in response to symptoms^12, 30^. Differences in testing frequency across cohorts could otherwise lead to differential detection of infections if routine testing practices varied by exposure group.

Vaccine effectiveness, together with the corresponding 95% CI, was calculated from the aHR as 1–aHR when the aHR was <1 and as 1/aHR–1 when the aHR was ≥1^23, 65^, yielding a symmetric scale ranging from −100% to 100% to facilitate intuitive interpretation of both positive and negative effectiveness^23, 65^. Effectiveness over time was further assessed using 3-month intervals following vaccination, with aHRs estimated for each interval using separate Cox regression models and failures restricted to the corresponding time intervals.

In subgroup analyses with sparse outcome data resulting in non-informative risk sets, standard Cox regression was replaced with Firth-penalized Cox proportional hazards models to reduce small-sample bias and improve estimate stability, with 95% CIs derived using the profile-likelihood method^67, 68^. These analyses were implemented using the *coxphf* package in R version 4.5.2 (R Core Team, Vienna, Austria).

The proportional hazards assumption was assessed using Schoenfeld residuals and log-log plots for each Cox regression model. Departures from the proportional hazards assumption were generally observed across models. Such departures are expected in the presence of waning immunity and thus time-varying effects during follow-up^69^. In this context, hazard ratios were interpreted as weighted averages of time-varying effects over the follow-up period, rather than as constant effects^69^. Accordingly, vaccine effectiveness estimates derived from these models represent average effectiveness over time^69^.

To further characterize time-varying effects, analyses were conducted within 3-month intervals following vaccination^69^. These interval-specific estimates provide insight into time-since-vaccination patterns over shorter follow-up periods, during which violations of the proportional hazards assumption are less likely and, if present, are of smaller magnitude.

# **Figure S6. Daily counts of second-dose vaccinations for A) BNT162b2 and B) mRNA-1273 during the study period, from 1 January 2021 to 18 December 2021.**

Alt text: Two-panel clustered-column charts showing second-dose vaccination scale-up. BNT162b2 second doses increase earlier in 2021, while mRNA-1273 second doses rise later, reflecting staggered vaccine roll-out.

# **Table S1. Characteristics of the eligible unmatched cases and controls in the BNT162b2 and mRNA-1273 analyses of the test-negative case-control study.**

| Characteristics | BNT162b2 analysis | | | mRNA-1273 analysis | | |
| --- | --- | --- | --- | --- | --- | --- |
|  | **Cases^*^** | **Controls^*^** | **SMD^†^** | **Cases^*^** | **Controls^*^** | **SMD^†^** |
|  | **N=218,179** | **N=4,937,013** |  | **N=205,708** | **N=4,053,241** |  |
| Median age (IQR) – years | 32 (24-40) | 32 (24-41) | 0.03^‡^ | 32 (24-39) | 31 (23-39) | 0.03^‡^ |
| Age group – n (%) |  |  |  |  |  |  |
| <10 years | 21,253 (9.7) | 496,307 (10.1) | 0.10 | 21,253 (10.3) | 496,299 (12.2) | 0.10 |
| 10-19 years | 19,563 (9.0) | 419,359 (8.5) |  | 18,556 (9.0) | 307,308 (7.6) |  |
| 20-29 years | 47,643 (21.8) | 1,119,603 (22.7) |  | 45,974 (22.3) | 979,277 (24.2) |  |
| 30-39 years | 73,796 (33.8) | 1,547,998 (31.4) |  | 70,012 (34.0) | 1,311,918 (32.4) |  |
| 40-49 years | 38,257 (17.5) | 824,903 (16.7) |  | 35,296 (17.2) | 642,720 (15.9) |  |
| 50-59 years | 12,993 (6.0) | 373,360 (7.6) |  | 11,287 (5.5) | 236,564 (5.8) |  |
| 60-69 years | 3,523 (1.6) | 123,052 (2.5) |  | 2,540 (1.2) | 62,748 (1.5) |  |
| 70+ years | 1,151 (0.5) | 32,431 (0.7) |  | 790 (0.4) | 16,407 (0.4) |  |
| Sex |  |  |  |  |  |  |
| Male | 147,702 (67.7) | 3,474,430 (70.4) | 0.06 | 140,474 (68.3) | 2,932,970 (72.4) | 0.09 |
| Female | 70,477 (32.3) | 1,462,583 (29.6) |  | 65,234 (31.7) | 1,120,271 (27.6) |  |
| Nationality^§^ |  |  |  |  |  |  |
| Bangladeshi | 15,154 (6.9) | 243,643 (4.9) | 0.28 | 14,937 (7.3) | 242,437 (6.0) | 0.25 |
| Egyptian | 12,444 (5.7) | 245,473 (5.0) |  | 11,307 (5.5) | 196,258 (4.8) |  |
| Filipino | 23,468 (10.8) | 276,137 (5.6) |  | 22,842 (11.1) | 239,757 (5.9) |  |
| Indian | 58,931 (27.0) | 1,427,519 (28.9) |  | 57,207 (27.8) | 1,317,312 (32.5) |  |
| Nepalese | 18,065 (8.3) | 325,703 (6.6) |  | 17,956 (8.7) | 329,899 (8.1) |  |
| Pakistani | 10,684 (4.9) | 241,800 (4.9) |  | 10,298 (5.0) | 224,115 (5.5) |  |
| Qatari | 26,254 (12.0) | 809,072 (16.4) |  | 21,666 (10.5) | 394,346 (9.7) |  |
| Sri Lankan | 6,860 (3.1) | 99,403 (2.0) |  | 6,767 (3.3) | 93,832 (2.3) |  |
| Sudanese | 5,646 (2.6) | 96,957 (2.0) |  | 5,294 (2.6) | 79,438 (2.0) |  |
| Other nationalities^‖^ | 40,673 (18.6) | 1,171,306 (23.7) |  | 37,434 (18.2) | 935,847 (23.1) |  |
| Coexisting conditions |  |  |  |  |  |  |
| 0 | 176,070 (80.7) | 4,114,434 (83.3) | 0.08 | 168,887 (82.1) | 3,565,684 (88.0) | 0.17 |
| 1 | 24,575 (11.3) | 464,226 (9.4) |  | 22,404 (10.9) | 311,335 (7.7) |  |
| 2 | 9,801 (4.5) | 181,647 (3.7) |  | 8,562 (4.2) | 103,060 (2.5) |  |
| 3 | 3,665 (1.7) | 79,322 (1.6) |  | 2,973 (1.4) | 36,545 (0.9) |  |
| 4 | 1,907 (0.9) | 44,839 (0.9) |  | 1,430 (0.7) | 17,797 (0.4) |  |
| 5 | 1,105 (0.5) | 25,776 (0.5) |  | 787 (0.4) | 9,474 (0.2) |  |
| 6+ | 1,056 (0.5) | 26,769 (0.5) |  | 665 (0.3) | 9,346 (0.2) |  |
| Reason for testing |  |  |  |  |  |  |
| Clinical suspicion^¶^ | 64,974 (29.8) | 282,186 (5.7) | 1.10 | 60,836 (29.6) | 222,538 (5.5) | 1.10 |
| Contact tracing | 26,166 (12.0) | 157,811 (3.2) |  | 25,112 (12.2) | 143,990 (3.6) |  |
| Port of entry | 46,484 (21.3) | 2,141,406 (43.4) |  | 44,440 (21.6) | 1,901,865 (46.9) |  |
| Individual request | 15,761 (7.2) | 245,863 (5.0) |  | 14,886 (7.2) | 206,969 (5.1) |  |
| Survey | 36,472 (16.7) | 736,249 (14.9) |  | 34,266 (16.7) | 590,572 (14.6) |  |
| Healthcare routine testing | 18,805 (8.6) | 158,850 (3.2) |  | 18,450 (9.0) | 128,591 (3.2) |  |
| Pre-travel | 8,817 (4.0) | 1,201,148 (24.3) |  | 7,063 (3.4) | 849,237 (21.0) |  |
| Other | 700 (0.3) | 13,498 (0.3) |  | 655 (0.3) | 9,476 (0.2) |  |
| Missing | 0 (0.0) | 2 (0.0) |  | 0 (0.0) | 3 (0.0) |  |

IQR denotes interquartile range, SARS-CoV-2, severe acute respiratory syndrome coronavirus 2, and SMD, standardized mean difference.

^*^Cases represent SARS-CoV-2-positive tests and controls represent SARS-CoV-2-negative tests.

^†^SMD is the difference in the mean of a covariate between groups divided by the pooled standard deviation. An SMD of ≤0.1 indicates adequate matching.

^‡^SMD is for the mean difference between groups divided by the pooled standard deviation.

^§^Nationalities were chosen to represent the most populous groups in Qatar.

^‖^These comprise up to 183 other nationalities in Qatar.

^¶^The tests used to define symptomatic infection.

# **Table S2. Baseline characteristics of the eligible unmatched vaccinated and unvaccinated cohorts in the BNT162b2 and mRNA-1273 analyses of the retrospective cohort study.**

| **Characteristics** | **BNT162b2 analysis** | | | **mRNA-1273 analysis** | | |
| --- | --- | --- | --- | --- | --- | --- |
|  | **Two-dose cohort** | **Unvaccinated cohort** | **SMD^*^** | **Two-dose cohort** | **Unvaccinated cohort** | **SMD^*^** |
|  | **N=1,122,116** | **N=2,461,352** |  | **N=780,479** | **N=2,461,352** |  |
| **Median age (IQR)** – **years** | 39 (31-47) | 32 (25-40) | 0.58**^†^** | 38 (33-45) | 32 (25-40) | 0.63**^†^** |
| **Age group** – **n (%)** |  |  |  |  |  |  |
| <10 years | 5 (0.0) | 231,350 (9.4) | 0.64 | 0 (0.0) | 231,350 (9.4) | 0.77 |
| 10-19 years | 73,972 (6.6) | 179,831 (7.3) |  | 12 (0.0) | 179,831 (7.3) |  |
| 20-29 years | 149,834 (13.4) | 577,137 (23.4) |  | 98,402 (12.6) | 577,137 (23.4) |  |
| 30-39 years | 353,727 (31.5) | 808,716 (32.9) |  | 343,591 (44.0) | 808,716 (32.9) |  |
| 40-49 years | 317,475 (28.3) | 421,679 (17.1) |  | 224,189 (28.7) | 421,679 (17.1) |  |
| 50-59 years | 145,892 (13.0) | 172,018 (7.0) |  | 90,107 (11.5) | 172,018 (7.0) |  |
| 60-69 years | 59,437 (5.3) | 55,159 (2.2) |  | 20,581 (2.6) | 55,159 (2.2) |  |
| 70+ years | 21,774 (1.9) | 15,462 (0.6) |  | 3,597 (0.5) | 15,462 (0.6) |  |
| **Sex** |  |  |  |  |  |  |
| Male | 781,854 (69.7) | 1,742,828 (70.8) | 0.02 | 632,252 (81.0) | 1,742,828 (70.8) | 0.24 |
| Female | 340,262 (30.3) | 718,524 (29.2) |  | 148,227 (19.0) | 718,524 (29.2) |  |
| **Nationality**^‡^ |  |  |  |  |  |  |
| Bangladeshi | 124,304 (11.1) | 175,096 (7.1) | 0.26 | 151,721 (19.4) | 175,096 (7.1) | 0.59 |
| Egyptian | 62,142 (5.5) | 132,633 (5.4) |  | 30,356 (3.9) | 132,633 (5.4) |  |
| Filipino | 100,941 (9.0) | 159,557 (6.5) |  | 69,087 (8.9) | 159,557 (6.5) |  |
| Indian | 249,925 (22.3) | 716,130 (29.1) |  | 215,631 (27.6) | 716,130 (29.1) |  |
| Nepalese | 95,579 (8.5) | 212,388 (8.6) |  | 108,968 (14.0) | 212,388 (8.6) |  |
| Pakistani | 47,663 (4.2) | 129,131 (5.2) |  | 41,724 (5.3) | 129,131 (5.2) |  |
| Qatari | 157,060 (14.0) | 234,601 (9.5) |  | 14,351 (1.8) | 234,601 (9.5) |  |
| Sri Lankan | 34,305 (3.1) | 68,714 (2.8) |  | 32,039 (4.1) | 68,714 (2.8) |  |
| Sudanese | 24,780 (2.2) | 51,791 (2.1) |  | 13,399 (1.7) | 51,791 (2.1) |  |
| Other nationalities^§^ | 225,417 (20.1) | 581,311 (23.6) |  | 103,203 (13.2) | 581,311 (23.6) |  |
| **Coexisting conditions** |  |  |  |  |  |  |
| 0 | 892,609 (79.5) | 2,123,530 (86.3) | 0.20 | 701,682 (89.9) | 2,123,530 (86.3) | 0.12 |
| 1 | 112,435 (10.0) | 195,045 (7.9) |  | 44,057 (5.6) | 195,045 (7.9) |  |
| 2 | 54,284 (4.8) | 74,828 (3.0) |  | 20,010 (2.6) | 74,828 (3.0) |  |
| 3 | 26,885 (2.4) | 31,301 (1.3) |  | 7,914 (1.0) | 31,301 (1.3) |  |
| 4 | 16,349 (1.5) | 17,126 (0.7) |  | 3,689 (0.5) | 17,126 (0.7) |  |
| 5 | 9,633 (0.9) | 9,686 (0.4) |  | 1,791 (0.2) | 9,686 (0.4) |  |
| 6+ | 9,921 (0.9) | 9,836 (0.4) |  | 1,336 (0.2) | 9,836 (0.4) |  |

IQR denotes interquartile range and SMD, standardized mean difference.

^*^SMD is the difference in the mean of a covariate between groups divided by the pooled standard deviation. An SMD of ≤0.1 indicates adequate matching.

^†^SMD is for the mean difference between groups divided by the pooled standard deviation.

^‡^Nationalities were chosen to represent the most populous groups in Qatar.

^§^These comprise up to 183 other nationalities in Qatar.

# **Figure S7. Vaccine effectiveness at 3-month intervals following primary series vaccination with A) BNT162b2 and B) mRNA-1273 in the test-negative case-control study. Effectiveness is shown against any SARS-CoV-2 infection, symptomatic infection, and severe, critical, or fatal COVID-19.**

COVID-19 denotes coronavirus disease and SARS-CoV-2, severe acute respiratory syndrome coronavirus 2.

^*^A symptomatic infection was defined as a SARS-CoV-2-positive test conducted because of the presence of symptoms consistent with a respiratory tract infection.

^†^Severity, criticality, and fatality were defined according to the World Health Organization guidelines.

^‡^The 95% CI was estimated with the use of McNemar’s test because of zero events among exposed cases.

Alt text: Two-panel dot-and-whisker plots showing waning vaccine effectiveness after primary series vaccination. Protection against infection outcomes declines rapidly over successive 3-month intervals, while protection against severe outcomes remains high.

# **Figure S8. Vaccine effectiveness at 3-month intervals following primary series vaccination with BNT162b2 in the retrospective cohort study A) without adjustment for testing rate and B) with adjustment for testing rate. Effectiveness is shown against any SARS-CoV-2 infection, symptomatic infection, and severe, critical, or fatal COVID-19.**

COVID-19 denotes coronavirus disease and SARS-CoV-2, severe acute respiratory syndrome coronavirus 2.

^*^A symptomatic infection was defined as a SARS-CoV-2-positive test conducted because of the presence of symptoms consistent with a respiratory tract infection.

^†^Severity, criticality, and fatality were defined according to the World Health Organization guidelines.

Alt text: Two-panel dot-and-whisker plots showing waning BNT162b2 effectiveness after primary series vaccination. Protection against infection outcomes declines rapidly over successive 3-month intervals, while protection against severe outcomes remains high. Adjustment for testing rate raises infection-outcome estimates.

# **Figure S9. Vaccine effectiveness at 3-month intervals following primary series vaccination with mRNA-1273 in the retrospective cohort study A) without adjustment for testing rate and B) with adjustment for testing rate. Effectiveness is shown against any SARS-CoV-2 infection, symptomatic infection, and severe, critical, or fatal COVID-19.**

COVID-19 denotes coronavirus disease and SARS-CoV-2, severe acute respiratory syndrome coronavirus 2.

^*^A symptomatic infection was defined as a SARS-CoV-2-positive test conducted because of the presence of symptoms consistent with a respiratory tract infection.

^†^Severity, criticality, and fatality were defined according to the World Health Organization guidelines.

^‡^Cox proportional hazards models could not be estimated due to the extremely small number of outcome events relative to cohort size, resulting in non-informative risk sets. Hazard ratios were estimated using Firth-penalized Cox proportional hazards regression to ensure stable estimation in the presence of sparse outcome data. CIs were derived using the profile-likelihood method. The negative lower bound for the confidence interval was truncated because the confidence interval was too wide.

Alt text: Two-panel dot-and-whisker plots showing waning mRNA-1273 effectiveness after primary series vaccination. Protection against infection outcomes declines rapidly over successive 3-month intervals, while protection against severe outcomes remains high. Adjustment for testing rate raises infection-outcome estimates.

# **Table S3. Additional analysis restricting the test-negative design to SARS-CoV-2-positive and -negative tests occurring during cohort study follow-up for primary-series BNT162b2 and mRNA-1273 vaccination, against any SARS-CoV-2 infection, symptomatic infection, and severe, critical, or fatal COVID-19.**

| **Effectiveness against** | **Cases^*^**  **(SARS-CoV-2-positive tests)** | | **Controls^*^**  **(SARS-CoV-2-negative tests)** | | **Effectiveness**  **% (95% CI)** |
| --- | --- | --- | --- | --- | --- |
|  | **Vaccinated** | **Unvaccinated** | **Vaccinated** | **Unvaccinated** |  |
| **BNT162b2 analysis** | | | | | |
| Any SARS-CoV-2 infection | 234 | 1,538 | 2,754 | 4,249 | 80.8  (77.4 to 83.6) |
| Symptomatic infection^†^ | 57 | 560 | 841 | 1,410 | 86.6  (81.3 to 90.3) |
| Severe, critical, or fatal COVID-19^‡^ | 2 | 117 | 138 | 231 | 97.0  (87.5 to 99.3) |
| **mRNA-1273 analysis** | | | | | |
| Any SARS-CoV-2 infection | 59 | 303 | 609 | 821 | 77.1  (68.3 to 83.4) |
| Symptomatic infection^†^ | 15 | 91 | 133 | 194 | 75.8  (55.1 to 86.9) |
| Severe, critical, or fatal COVID-19^‡^ | 0 | 21 | 29 | 37 | 100.0  (86.4 to 100.0)^§^ |

CI denotes confidence interval, COVID-19, coronavirus disease, and SARS-CoV-2, severe acute respiratory syndrome coronavirus 2.

^*^Cases and controls were selected from matched samples of the retrospective cohort design and classified as cases or controls according to positive or negative test results during follow-up. Cases and controls were matched exactly one-to-five to increase statistical power, given the low number of events. Not all cases could be matched to exactly five eligible controls; all matched sets with at least one eligible control were retained in the analysis. Matching was done by sex, 10-year age group, nationality, number of coexisting conditions, calendar week of testing, and reason for testing. Vaccine effectiveness estimates were based on odds ratios obtained from conditional logistic regressions.

^†^A symptomatic infection was defined as a SARS-CoV-2-positive test conducted because of the presence of symptoms consistent with a respiratory tract infection.

^‡^Severity, criticality, and fatality were defined according to the World Health Organization guidelines.

^§^The 95% CI was estimated with the use of McNemar’s test because of zero events among exposed cases.

# **Table S4. Strengthening the Reporting of Observational Studies in Epidemiology (STROBE) checklist for case-control studies.**

|  | Item No | Recommendation | Section |
| --- | --- | --- | --- |
| **Title and abstract** | 1 | (*a*) Indicate the study’s design with a commonly used term in the title or the abstract | Abstract |
|  |  | (*b*) Provide in the abstract an informative and balanced summary of what was done and what was found | Abstract |
| Introduction | | |  |
| Background/rationale | 2 | Explain the scientific background and rationale for the investigation being reported | Introduction & Supplementary Section S1 |
| Objectives | 3 | State specific objectives, including any prespecified hypotheses | Introduction & Supplementary Section S1 |
| Methods | | |  |
| Study design | 4 | Present key elements of study design | Methods (‘Study design’) & Supplementary Section S6 |
| Setting | 5 | Describe the setting, locations, and relevant dates, including periods of recruitment, exposure, follow-up, and data collection | Methods (‘Study population and data sources’ & ‘Study design’) & Supplementary Sections S2-S3 & S6 |
| Participants | 6 | (*a*) Give the eligibility criteria, and the sources and methods of case ascertainment and control selection. Give the rationale for the choice of cases and controls | Methods (‘Study population and data sources’ & ‘Study design’), Supplementary Section S6, & Supplementary Figures S2 & S3 |
|  |  | (*b*) For matched studies, give matching criteria and the number of controls per case |  |
| Variables | 7 | Clearly define all outcomes, exposures, predictors, potential confounders, and effect modifiers. Give diagnostic criteria, if applicable | Methods (‘Study design’) & Supplementary Sections S2-S6 |
| Data sources/ measurement | 8 | For each variable of interest, give sources of data and details of methods of assessment (measurement). Describe comparability of assessment methods if there is more than one group | Methods (‘Study population and data sources’ & ‘Study design’) & Supplementary Sections S2-S6 |
| Bias | 9 | Describe any efforts to address potential sources of bias | Methods (‘Study design’) & Supplementary Sections S6 & S8 |
| Study size | 10 | Explain how the study size was arrived at | Supplementary Figures S2 & S3 |
| Quantitative variables | 11 | Explain how quantitative variables were handled in the analyses. If applicable, describe which groupings were chosen and why | Methods (‘Study design’), Supplementary Sections S6 & S8 |
| Statistical methods | 12 | (*a*) Describe all statistical methods, including those used to control for confounding | Methods (‘Study design’) & Supplementary Sections S6 & S8 |
|  |  | (*b*) Describe any methods used to examine subgroups and interactions | Methods (‘Study design’) & Supplementary Sections S6 & S8 |
|  |  | (*c*) Explain how missing data were addressed | Not applicable, see Methods (‘Study population and data sources’) & Supplementary Section S2 |
|  |  | (*d*) If applicable, explain how matching of cases and controls was addressed | Methods (‘Study design’) & Supplementary Sections S6 & S8 |
|  |  | (*e*) Describe any sensitivity analyses | Not applicable |
| Results | | |  |
| Participants | 13 | (a) Report numbers of individuals at each stage of study—eg numbers potentially eligible, examined for eligibility, confirmed eligible, included in the study, completing follow-up, and analysed | Table 1, Supplementary Figures S2 & S3, & Supplementary Table S1 |
|  |  | (b) Give reasons for non-participation at each stage |  |
|  |  | (c) Consider use of a flow diagram |  |
| Descriptive data | 14 | (a) Give characteristics of study participants (eg demographic, clinical, social) and information on exposures and potential confounders | Results (‘Study population’), Table 1, & Supplementary Table S1 |
|  |  | (b) Indicate number of participants with missing data for each variable of interest | Not applicable, see Methods (‘Study population and data sources’) & Supplementary Section S2 |
| Outcome data | 15 | Report numbers in each exposure category, or summary measures of exposure | Results (‘Vaccine effectiveness’), Table 3, & Figure 1 |
| Main results | 16 | (*a*) Give unadjusted estimates and, if applicable, confounder-adjusted estimates and their precision (eg, 95% confidence interval). Make clear which confounders were adjusted for and why they were included | Results (‘Vaccine effectiveness’), Table 3, & Figure 1 |
|  |  | (*b*) Report category boundaries when continuous variables were categorized | Table 1 & Supplementary Table S1 |
|  |  | (*c*) If relevant, consider translating estimates of relative risk into absolute risk for a meaningful time period | Not applicable |
| Other analyses | 17 | Report other analyses done—eg analyses of subgroups and interactions, and sensitivity analyses | Results (‘Vaccine effectiveness’ & ‘Additional analysis’), Figure 3, Supplementary Figure S7, and Supplementary Table S3 |
| Discussion | | |  |
| Key results | 18 | Summarise key results with reference to study objectives | Discussion, paragraphs 1-7 |
| Limitations | 19 | Discuss limitations of the study, taking into account sources of potential bias or imprecision. Discuss both direction and magnitude of any potential bias | Discussion, paragraphs 8-9 & Supplementary Section S6 |
| Interpretation | 20 | Give a cautious overall interpretation of results considering objectives, limitations, multiplicity of analyses, results from similar studies, and other relevant evidence | Discussion, paragraphs 1-7 and 11 |
| Generalisability | 21 | Discuss the generalisability (external validity) of the study results | Discussion, paragraph 8 |
| Other information | | |  |
| Funding | 22 | Give the source of funding and the role of the funders for the present study and, if applicable, for the original study on which the present article is based | Funding & Acknowledgements |

# **Table S5. Strengthening the Reporting of Observational Studies in Epidemiology (STROBE) checklist for cohort studies.**

|  | Item No | Recommendation | Main Text page |
| --- | --- | --- | --- |
| **Title and abstract** | 1 | (*a*) Indicate the study’s design with a commonly used term in the title or the abstract | Abstract |
|  |  | (*b*) Provide in the abstract an informative and balanced summary of what was done and what was found |  |
| Introduction | | | |
| Background/rationale | 2 | Explain the scientific background and rationale for the investigation being reported | Introduction & Supplementary Section S1 |
| Objectives | 3 | State specific objectives, including any prespecified hypotheses | Introduction & Supplementary Section S1 |
| Methods | | | |
| Study design | 4 | Present key elements of study design early in the paper | Methods (‘Study design’) & Supplementary Section S7 |
| Setting | 5 | Describe the setting, locations, and relevant dates, including periods of recruitment, exposure, follow-up, and data collection | Methods (‘Study population and data sources’ & ‘Study design’) & Supplementary Sections S2-S3 & S7 |
| Participants | 6 | (*a*) Give the eligibility criteria, and the sources and methods of selection of participants. Describe methods of follow-up | Methods (‘Study population and data sources’ & ‘Study design’), Supplementary Section S7, & Supplementary Figures S4 & S5 |
|  |  | (*b*) For matched studies, give matching criteria and number of exposed and unexposed |  |
| Variables | 7 | Clearly define all outcomes, exposures, predictors, potential confounders, and effect modifiers. Give diagnostic criteria, if applicable | Methods (‘Study design’) & Supplementary Sections S2-S5 & S7 |
| Data sources/ measurement | 8 | For each variable of interest, give sources of data and details of methods of assessment (measurement). Describe comparability of assessment methods if there is more than one group | Methods (‘Study population and data sources’ & ‘Study design’) Supplementary Sections S2-S5 & S7 |
| Bias | 9 | Describe any efforts to address potential sources of bias | Methods (‘Study design’) & Supplementary Sections S7 & S8 |
| Study size | 10 | Explain how the study size was arrived at | Supplementary Figures S4 & S5 |
| Quantitative variables | 11 | Explain how quantitative variables were handled in the analyses. If applicable, describe which groupings were chosen and why | Methods (‘Study design’), Supplementary Sections S7 & S8 |
| Statistical methods | 12 | (*a*) Describe all statistical methods, including those used to control for confounding | Methods (‘Study design’) & Supplementary Sections S7 & S8 |
|  |  | (*b*) Describe any methods used to examine subgroups and interactions | Methods (‘Study design’) & Supplementary Sections S7 & S8 |
|  |  | (*c*) Explain how missing data were addressed | Not applicable, see Methods (‘Study population and data sources’) & Supplementary Section S2 |
|  |  | (*d*) If applicable, explain how loss to follow-up was addressed | Methods (‘Study design’) & Supplementary Sections S7 & S8 |
|  |  | (*e*) Describe any sensitivity analyses | Not applicable |
| Results | | |  |
| Participants | 13 | (a) Report numbers of individuals at each stage of study—eg numbers potentially eligible, examined for eligibility, confirmed eligible, included in the study, completing follow-up, and analysed | Table 2, Supplementary Figures S4 & S5, & Supplementary Table S2 |
|  |  | (b) Give reasons for non-participation at each stage |  |
|  |  | (c) Consider use of a flow diagram |  |
| Descriptive data | 14 | (a) Give characteristics of study participants (eg demographic, clinical, social) and information on exposures and potential confounders | Results (‘Study population’), Table 2, & Supplementary Table S2 |
|  |  | (b) Indicate number of participants with missing data for each variable of interest | Not applicable, see Methods (‘Study population and data sources’) & Supplementary Section S2 |
|  |  | (c) Summarise follow-up time (eg, average and total amount) | Results (‘Vaccine effectiveness’), Figures 1 & 2, Table 4 |
| Outcome data | 15 | Report numbers of outcome events or summary measures over time | Results (‘Vaccine effectiveness’), Figures 1 & 2, Table 4 |
| Main results | 16 | (a) Give unadjusted estimates and, if applicable, confounder-adjusted estimates and their precision (eg, 95% confidence interval). Make clear which confounders were adjusted for and why they were included | Results (‘Vaccine effectiveness’), Figures 1 & 2, Table 4 |
|  |  | (b) Report category boundaries when continuous variables were categorized | Table 2 & Supplementary Table S2 |
|  |  | (c) If relevant, consider translating estimates of relative risk into absolute risk for a meaningful time period | Not applicable |
| Other analyses | 17 | Report other analyses done—eg analyses of subgroups and interactions, and sensitivity analyses | Results (‘Vaccine effectiveness’ & ‘Additional analysis’), Figure 3, Supplementary Figures S8 & S9, and Supplementary Table S3 |
| **Discussion** | | | |
| Key results | 18 | Summarise key results with reference to study objectives | Discussion, paragraphs 1-7 |
| Limitations | 19 | Discuss limitations of the study, taking into account sources of potential bias or imprecision. Discuss both direction and magnitude of any potential bias | Discussion, paragraphs 8-9 & Supplementary Section S7 |
| Interpretation | 20 | Give a cautious overall interpretation of results considering objectives, limitations, multiplicity of analyses, results from similar studies, and other relevant evidence | Discussion, paragraphs 1-7 and 11 |
| Generalisability | 21 | Discuss the generalisability (external validity) of the study results | Discussion, paragraph 8 |
| **Other information** | | | |
| Funding | 22 | Give the source of funding and the role of the funders for the present study and, if applicable, for the original study on which the present article is based | Funding & Acknowledgements |

# **References**

1.	Rothwell PM. External validity of randomised controlled trials: "to whom do the results of this trial apply?". *Lancet* 2005; **365**: 82-93.

2.	Bothwell LE, Greene JA, Podolsky SH, Jones DS. Assessing the Gold Standard--Lessons from the History of RCTs. *N Engl J Med* 2016; **374**: 2175-81.

3.	Janiaud P, Hemkens LG, Ioannidis JPA. Challenges and Lessons Learned From COVID-19 Trials: Should We Be Doing Clinical Trials Differently? *Can J Cardiol* 2021; **37**: 1353-64.

4.	Chambers C. Using observational epidemiology to evaluate COVID-19 vaccines: integrating traditional methods with new data sources and tools. *Can J Public Health* 2021; **112**: 867-71.

5.	de Waure C, Gartner BC, Lopalco PL, Puig-Barbera J, Nguyen-Van-Tam JS. Real world evidence for public health decision-making on vaccination policies: perspectives from an expert roundtable. *Expert Rev Vaccines* 2024; **23**: 27-38.

6.	Feikin DR, Higdon MM, Abu-Raddad LJ, et al. Duration of effectiveness of vaccines against SARS-CoV-2 infection and COVID-19 disease: results of a systematic review and meta-regression. *Lancet* 2022; **399**: 924-44.

7.	Abu-Raddad LJ, Chemaitelly H, Ayoub HH, et al. Effect of mRNA Vaccine Boosters against SARS-CoV-2 Omicron Infection in Qatar. *N Engl J Med* 2022; **386**: 1804-16.

8.	Hernan MA, Robins JM. Using Big Data to Emulate a Target Trial When a Randomized Trial Is Not Available. *Am J Epidemiol* 2016; **183**: 758-64.

9.	Wang SV, Schneeweiss S, Initiative R-D, et al. Emulation of Randomized Clinical Trials With Nonrandomized Database Analyses: Results of 32 Clinical Trials. *JAMA* 2023; **329**: 1376-85.

10.	Jackson ML, Nelson JC. The test-negative design for estimating influenza vaccine effectiveness. *Vaccine* 2013; **31**: 2165-8.

11.	Verani JR, Baqui AH, Broome CV, et al. Case-control vaccine effectiveness studies: Preparation, design, and enrollment of cases and controls. *Vaccine* 2017; **35**: 3295-302.

12.	Chemaitelly H, Tang P, Hasan MR, et al. Waning of BNT162b2 Vaccine Protection against SARS-CoV-2 Infection in Qatar. *N Engl J Med* 2021; **385**: e83.

13.	Nelson JC, Jackson ML, Weiss NS, Jackson LA. New strategies are needed to improve the accuracy of influenza vaccine effectiveness estimates among seniors. *J Clin Epidemiol* 2009; **62**: 687-94.

14.	Chemaitelly H, Ayoub HH, Coyle P, et al. Assessing healthy vaccinee effect in COVID-19 vaccine effectiveness studies: a national cohort study in Qatar. *Elife* 2025; **14**.

15.	Bodner K, Wang L, Kustra R, et al. Impact of unequal testing on vaccine effectiveness estimates across two study designs: a simulation study. *Nat Commun* 2025; **16**: 4849.

16.	Li G, Gerlovin H, Figueroa Muniz MJ, et al. Comparison of the Test-negative Design and Cohort Design With Explicit Target Trial Emulation for Evaluating COVID-19 Vaccine Effectiveness. *Epidemiology* 2024; **35**: 137-49.

17.	Dickerman BA, Garcia-Albeniz X, Logan RW, Denaxas S, Hernan MA. Avoidable flaws in observational analyses: an application to statins and cancer. *Nat Med* 2019; **25**: 1601-6.

18.	Sullivan SG, Tchetgen Tchetgen EJ, Cowling BJ. Theoretical Basis of the Test-Negative Study Design for Assessment of Influenza Vaccine Effectiveness. *Am J Epidemiol* 2016; **184**: 345-53.

19.	Abu-Raddad LJ, Chemaitelly H, Butt AA, National Study Group for COVID-19 Vaccination. Effectiveness of the BNT162b2 Covid-19 Vaccine against the B.1.1.7 and B.1.351 Variants. *N Engl J Med* 2021; **385**: 187-9.

20.	Chemaitelly H, Yassine HM, Benslimane FM, et al. mRNA-1273 COVID-19 vaccine effectiveness against the B.1.1.7 and B.1.351 variants and severe COVID-19 disease in Qatar. *Nat Med* 2021; **27**: 1614-21.

21.	Altarawneh HN, Chemaitelly H, Hasan MR, et al. Protection against the Omicron Variant from Previous SARS-CoV-2 Infection. *N Engl J Med* 2022; **386**: 1288-90.

22.	Roltgen K, Nielsen SCA, Silva O, et al. Immune imprinting, breadth of variant recognition, and germinal center response in human SARS-CoV-2 infection and vaccination. *Cell* 2022; **185**: 1025-40 e14.

23.	Chemaitelly H, Ayoub HH, Tang P, et al. Long-term COVID-19 booster effectiveness by infection history and clinical vulnerability and immune imprinting: a retrospective population-based cohort study. *Lancet Infect Dis* 2023; **23**: 816-27.

24.	Chemaitelly H, Ayoub HH, Tang P, et al. History of primary-series and booster vaccination and protection against Omicron reinfection. *Sci Adv* 2023; **9**: eadh0761.

25.	Chemaitelly H, Ayoub HH, Coyle P, et al. BNT162b2 Versus mRNA-1273 Vaccines: Comparative Analysis of Long-Term Protection Against SARS-CoV-2 Infection and Severe COVID-19 in Qatar. *Influenza Other Respir Viruses* 2024; **18**: e13357.

26.	Chemaitelly H, Ayoub HH, Bobrovitz N, et al. Immune histories and natural infection protection during the omicron era. *Commun Med (Lond)* 2025; **5**: 262.

27.	Abu-Raddad LJ, Chemaitelly H, Ayoub HH, et al. Introduction and expansion of the SARS-CoV-2 B.1.1.7 variant and reinfections in Qatar: A nationally representative cohort study. *Plos Med* 2021; **18**: e1003879.

28.	Chemaitelly H, Bertollini R, Abu-Raddad LJ, National Study Group for COVID-19 Epidemiology. Efficacy of Natural Immunity against SARS-CoV-2 Reinfection with the Beta Variant. *N Engl J Med* 2021; **385**: 2585-6.

29.	Tang P, Hasan MR, Chemaitelly H, et al. BNT162b2 and mRNA-1273 COVID-19 vaccine effectiveness against the SARS-CoV-2 Delta variant in Qatar. *Nat Med* 2021; **27**: 2136-43.

30.	Altarawneh HN, Chemaitelly H, Ayoub HH, et al. Effects of Previous Infection and Vaccination on Symptomatic Omicron Infections. *N Engl J Med* 2022; **387**: 21-34.

31.	Polack FP, Thomas SJ, Kitchin N, et al. Safety and Efficacy of the BNT162b2 mRNA Covid-19 Vaccine. *N Engl J Med* 2020; **383**: 2603-15.

32.	Baden LR, El Sahly HM, Essink B, et al. Efficacy and Safety of the mRNA-1273 SARS-CoV-2 Vaccine. *N Engl J Med* 2021; **384**: 403-16.

33.	Abu-Raddad LJ, Chemaitelly H, Bertollini R, National Study Group for COVID-19 Vaccination. Effectiveness of mRNA-1273 and BNT162b2 Vaccines in Qatar. *N Engl J Med* 2022; **386**: 799-800.

34.	Abu-Raddad LJ, Chemaitelly H, Ayoub HH, et al. Characterizing the Qatar advanced-phase SARS-CoV-2 epidemic. *Sci Rep* 2021; **11**: 6233.

35.	Chemaitelly H, Ayoub HH, Coyle P, et al. Differential protection against SARS-CoV-2 reinfection pre- and post-Omicron. *Nature* 2025; **639**: 1024-31.

36.	Vogels C, Fauver J, Grubaugh N. Multiplexed RT-qPCR to screen for SARS-COV-2 B.1.1.7, B.1.351, and P.1 variants of concern V.3. dx.doi.org/10.17504/protocols.io.br9vm966. 2021.

37.	National Project of Surveillance for Variants of Concern and Viral Genome Sequencing. *Qatar viral genome sequencing data. Data on randomly collected samples.* [*https://www.gisaid.org/phylodynamics/global/nextstrain/*](https://www.gisaid.org/phylodynamics/global/nextstrain/). 2021 [cited; Available from: <https://www.gisaid.org/phylodynamics/global/nextstrain/>

38.	Benslimane FM, Al Khatib HA, Al-Jamal O, et al. One Year of SARS-CoV-2: Genomic Characterization of COVID-19 Outbreak in Qatar. *Front Cell Infect Microbiol* 2021; **11**: 768883.

39.	Hasan MR, Kalikiri MKR, Mirza F, et al. Real-Time SARS-CoV-2 Genotyping by High-Throughput Multiplex PCR Reveals the Epidemiology of the Variants of Concern in Qatar. *Int J Infect Dis* 2021; **112**: 52-4.

40.	Saththasivam J, El-Malah SS, Gomez TA, et al. COVID-19 (SARS-CoV-2) outbreak monitoring using wastewater-based epidemiology in Qatar. *Sci Total Environ* 2021; **774**: 145608.

41.	El-Malah SS, Saththasivam J, Jabbar KA, et al. Application of human RNase P normalization for the realistic estimation of SARS-CoV-2 viral load in wastewater: A perspective from Qatar wastewater surveillance. *Environ Technol Innov* 2022; **27**: 102775.

42.	Chemaitelly H, Ayoub HH, AlMukdad S, et al. Duration of mRNA vaccine protection against SARS-CoV-2 Omicron BA.1 and BA.2 subvariants in Qatar. *Nat Commun* 2022; **13**: 3082.

43.	Qassim SH, Chemaitelly H, Ayoub HH, et al. Effects of BA.1/BA.2 subvariant, vaccination and prior infection on infectiousness of SARS-CoV-2 omicron infections. *J Travel Med* 2022; **29**.

44.	Altarawneh HN, Chemaitelly H, Ayoub HH, et al. Protective Effect of Previous SARS-CoV-2 Infection against Omicron BA.4 and BA.5 Subvariants. *N Engl J Med* 2022; **387**: 1620-2.

45.	Chemaitelly H, Tang P, Coyle P, et al. Protection against Reinfection with the Omicron BA.2.75 Subvariant. *N Engl J Med* 2023; **388**: 665-7.

46.	Chemaitelly H, Coyle P, Ben Kacem MA, et al. Protection of natural infection against reinfection with SARS-CoV-2 JN.1 variant. *J Travel Med* 2024; **31**.

47.	Al-Thani MH, Farag E, Bertollini R, et al. SARS-CoV-2 Infection Is at Herd Immunity in the Majority Segment of the Population of Qatar. *Open Forum Infect Dis* 2021; **8**: ofab221.

48.	World Health Organization (WHO). Living guidance for clinical management of COVID-19. Aavailable from: <https://www.who.int/publications/i/item/WHO-2019-nCoV-clinical-2021-2>. Accessed on: February 27, 2023. 2023.

49.	World Health Organization (WHO). International Guidelines for Certification and Classification (Coding) of COVID-19 as Cause of Death. Available from: <https://www.who.int/publications/m/item/international-guidelines-for-certification-and-classification-(coding)-of-covid-19-as-cause-of-death>. Accessed on: February 27, 2023. 2023.

50.	Sukik L, Chemaitelly H, Ayoub HH, et al. Effectiveness of two and three doses of COVID-19 mRNA vaccines against infection, symptoms, and severity in the pre-omicron era: A time-dependent gradient. *Vaccine* 2024; **42**: 3307-20.

51.	Lopez Bernal J, Andrews N, Gower C, et al. Effectiveness of Covid-19 Vaccines against the B.1.617.2 (Delta) Variant. *N Engl J Med* 2021; **385**: 585-94.

52.	Chemaitelly H, Ayoub HH, Faust JS, et al. Turning point in COVID-19 severity and fatality during the pandemic: a national cohort study in Qatar. *BMJ Public Health* 2023; **1**: e000479.

53.	Sukik L, Chemaitelly H, Ayoub HH, et al. Evaluating Hospital Admission Data as Indicators of COVID-19 Severity: A National Assessment in Qatar. *Open Forum Infect Dis* 2025; **12**: ofaf098.

54.	Abu-Raddad LJ, Chemaitelly H, Bertollini R, National Study Group for COVID-19 Vaccination. Waning mRNA-1273 Vaccine Effectiveness against SARS-CoV-2 Infection in Qatar. *N Engl J Med* 2022; **386**: 1091-3.

55.	Ayoub HH, Tomy M, Chemaitelly H, et al. Estimating protection afforded by prior infection in preventing reinfection: applying the test-negative study design. *Am J Epidemiol* 2024; **193**: 883-97.

56.	Coyle PV, Chemaitelly H, Ben Hadj Kacem MA, et al. SARS-CoV-2 seroprevalence in the urban population of Qatar: An analysis of antibody testing on a sample of 112,941 individuals. *iScience* 2021; **24**: 102646.

57.	Jeremijenko A, Chemaitelly H, Ayoub HH, et al. Herd Immunity against Severe Acute Respiratory Syndrome Coronavirus 2 Infection in 10 Communities, Qatar. *Emerg Infect Dis* 2021; **27**: 1343-52.

58.	Ayoub HH, Chemaitelly H, Seedat S, et al. Mathematical modeling of the SARS-CoV-2 epidemic in Qatar and its impact on the national response to COVID-19. *J Glob Health* 2021; **11**: 05005.

59.	Chemaitelly H, AlMukdad S, Ayoub HH, et al. Covid-19 Vaccine Protection among Children and Adolescents in Qatar. *N Engl J Med* 2022; **387**: 1865-76.

60.	Chemaitelly H, Ayoub HH, AlMukdad S, et al. Protection from previous natural infection compared with mRNA vaccination against SARS-CoV-2 infection and severe COVID-19 in Qatar: a retrospective cohort study. *Lancet Microbe* 2022; **3**: e944-e55.

61.	Barda N, Dagan N, Cohen C, et al. Effectiveness of a third dose of the BNT162b2 mRNA COVID-19 vaccine for preventing severe outcomes in Israel: an observational study. *Lancet* 2021; **398**: 2093-100.

62.	Kahn R, Schrag SJ, Verani JR, Lipsitch M. Identifying and Alleviating Bias Due to Differential Depletion of Susceptible People in Postmarketing Evaluations of COVID-19 Vaccines. *Am J Epidemiol* 2022; **191**: 800-11.

63.	Ray GT, Lewis N, Klein NP, Daley MF, Lipsitch M, Fireman B. Depletion-of-susceptibles Bias in Analyses of Intra-season Waning of Influenza Vaccine Effectiveness. *Clin Infect Dis* 2020; **70**: 1484-6.

64.	Austin PC. Using the Standardized Difference to Compare the Prevalence of a Binary Variable Between Two Groups in Observational Research. *Commun Stat-Simul C* 2009; **38**: 1228-34.

65.	Tseng HF, Ackerson BK, Bruxvoort KJ, et al. Effectiveness of mRNA-1273 vaccination against SARS-CoV-2 omicron subvariants BA.1, BA.2, BA.2.12.1, BA.4, and BA.5. *Nat Commun* 2023; **14**: 189.

66.	Sjolander A, Greenland S. Ignoring the matching variables in cohort studies - when is it valid and why? *Stat Med* 2013; **32**: 4696-708.

67.	Heinze G, Dunkler D. Avoiding infinite estimates of time-dependent effects in small-sample survival studies. *Stat Med* 2008; **27**: 6455-69.

68.	Heinze G, Schemper M. A solution to the problem of monotone likelihood in Cox regression. *Biometrics* 2001; **57**: 114-9.

69.	Stensrud MJ, Hernan MA. Why Test for Proportional Hazards? *JAMA* 2020; **323**: 1401-2.
